# Nucleoid and cytoplasmic localization of small RNAs in *Escherichia coli*

**DOI:** 10.1093/nar/gkx023

**Published:** 2017-01-24

**Authors:** Huanjie Sheng, Weston T. Stauffer, Razika Hussein, Chris Lin, Han N. Lim

**Affiliations:** Department of Integrative Biology, 3060 Valley Life Sciences Building, Mail code 3140, University of California, Berkeley, CA, 94720-3140, USA

## Abstract

Bacterial small RNAs (sRNAs) regulate protein production by binding to mRNAs and altering their translation and degradation. sRNAs are smaller than most mRNAs but larger than many proteins. Therefore it is uncertain whether sRNAs can enter the nucleoid to target nascent mRNAs. Here, we investigate the intracellular localization of sRNAs transcribed from plasmids in *Escherichia coli* using RNA fluorescent *in-situ* hybridization. We found that sRNAs (GlmZ, OxyS, RyhB and SgrS) have equal preference for the nucleoid and cytoplasm, and no preferential localization at the cell membrane. We show using the *gfp* mRNA (encoding green fluorescent protein) that non-sRNAs can be engineered to have different proportions of nucleoid and cytoplasmic localization by altering their length and/or translation. The same localization as sRNAs was achieved by decreasing *gfp* mRNA length and translation, which suggests that sRNAs and other RNAs may enter the densely packed DNA of the nucleoid if they are sufficiently small. We also found that the Hfq protein, which binds sRNAs, minimally affects sRNA localization. Important implications of our findings for engineering synthetic circuits are: (i) sRNAs can potentially bind nascent mRNAs in the nucleoid, and (ii) localization patterns and distribution volumes of sRNAs can differ from some larger RNAs.

## INTRODUCTION

Bacterial small RNAs (sRNAs) regulate the production of diverse classes of proteins in a wide variety of pathways (1). Most sRNAs bind to target mRNAs at or near the translation initiation region (TIR) and form sRNA-mRNA duplexes. Duplex formation commonly decreases translation and/or increases mRNA degradation resulting in decreased target protein production (1). Less often, duplex formation has the opposite effect, causing increased target protein production (1). Some sRNAs can decrease the production of target proteins and increase the production of others (2–7). Additionally, some sRNAs regulate gene expression by: binding directly to the σ^70^-RNA polymerase holoenzyme to alter transcription (8), sequestering proteins (9) and being translated into an active peptide (10).

Most sRNA regulation in *Escherichia coli* and in many other bacteria requires the Hfq protein (11). Hfq primarily exists as a hexamer that can bind sRNAs and mRNAs to alter their folding and/or facilitate duplex formation. In addition, Hfq can mediate the interaction of proteins and complexes (including RNase E, ribosomes, poly(A) polymerase I and polynucleotide phosphorylase) with sRNAs, mRNAs and/or duplexes (12,13). Hfq has been shown by electron microscopy to be present at the inner cell membrane, as well as in the nucleoid and cytoplasm (14). Many of the proteins that bind to Hfq are also found in the cytoplasm and/or at the cell membrane (15–17).

It has yet to be determined where sRNAs localize to in the cell, which is a barrier to understanding their mechanism of action and the constraints on their activity. It is often assumed that most RNAs are small, and thus they can move anywhere in the cell. However in actuality, RNAs are usually large compared to the proteins they encode due to: (i) each RNA having three nucleotides for each amino acid encoded (in addition there are 5΄ and 3΄ untranslated RNA sequences); (ii) the average nucleotide is three times the mass of an amino acid (≈330 Daltons and ≈110 Daltons respectively) (18); and (iii) RNAs often have less compact structures than globular proteins (18). Therefore even relatively short sRNAs are large compared to some of the small proteins that act as transcription factors. For example, the diameters of the MicA and DsrA sRNAs are approximately 87.5 Å and 111.5 Å (19) whereas the typical globular protein has a diameter of 50 Å (18). Therefore while transcription factors can move through the densely packed DNA of the nucleoid to bind near the promoters of target genes, it is possible that many sRNAs and mRNAs are not able to do so because of their larger size.

In general, factors other than size and structure can also affect RNA and protein localization, including: (i) the molecules they form complexes with, which can transport them (20,21) or restrict them (22) to specific sites in the cell; (ii) covalent modifications (23,24); and (iii) net electrostatic charge and charge distribution (25,26). In bacteria, a variety of RNA localization patterns and mechanisms have been reported. It has long been recognized that the signal recognition particle (SRP) pathway is an important mechanism for RNA and protein localization. SRP recognizes signal sequences at the N-terminal end of nascent proteins, leading to the transport of a complex containing the partial mRNA, ribosome, and partly synthesized protein (27) to the cell membrane [note: it has also been proposed that these components may be transported separately (28)]. Once at the cell membrane, translation continues in conjunction with translocation of the protein across the cell membrane (27). Recently, it has been demonstrated that mRNAs can also be transported to the cell membrane without being coupled to translation via a mechanism that has not been fully elucidated involving RNA zip codes (29). Bacteria also have mechanisms to localize RNAs to other cellular regions including the cytoplasm (29–31), cell poles (29,31) and septa of dividing cells (32). Other studies have shown that some mRNAs primarily localize to their site of transcription (33) (note: it is unclear whether this transcription is taking place at the edge or the center of the nucleoid). In summary, regulation of RNA localization is important to cells, there are diverse sites and complex patterns of localization, and multiple localization mechanisms, of which most are poorly understood.

Whether or not sRNAs can localize to the nucleoid has important implications for gene regulation. An inability of sRNAs to enter the nucleoid would prevent them binding the TIR on target mRNAs as soon as it is transcribed, and give ribosomes greater opportunity for assembling at the TIR and initiating translation. Consequently, sRNAs would only be able to bind the mRNA after the transcription-translation complex has formed and moved to either the outer edge of the nucleoid or the membrane (34,35). At the edge of the nucleoid, transcription and translation occur where there is a high concentration of ribosomes (36–40), which may make it more difficult for the sRNA and Hfq to compete for binding at the TIR. Localization has been reported for one sRNA-mRNA pair (SgrS-*ptsG* mRNA); and in this pair, translation of the transmembrane domain of the *ptsG* mRNA is required for SgrS to mediate degradation of this mRNA (34). Therefore in this case, it appears the sRNA does not need to enter the nucleoid to mediate its actions. Because *ptsG* requires at least one round of translation for transport to the membrane, SgrS cannot completely silence PtsG production (34). In theory, complete silencing of target protein production is achievable if sRNAs bind to target mRNAs soon after they are transcribed and before any translation is initiated. The 6S sRNA, which regulates gene transcription by binding to the RNA polymerase holoenzyme with the sigma70 factor (41), indicates that it is possible for at least some sRNAs to move through the nucleoid to sites where transcription is initiated.

Due to the fundamental roles of sRNAs in bacterial survival and pathogenesis, identification of their cellular localization will benefit many areas of basic and medical research. Knowledge of sRNA localization within cells will also aid the rational design and optimization of their use in engineered gene regulatory circuits. sRNAs are useful components in regulatory circuits because of their properties, including rapid signaling (42), programmable specificity (35,43) and threshold-linear responses (42,44). sRNAs have been used as tools to investigate the properties of gene regulation (42,44–46) and to construct circuits for metabolic engineering and ‘knock-down’ studies (47,48).

In this study we investigated whether sRNAs preferentially accumulate in the nucleoid, cytoplasm, or cell membrane using synthetic sRNA systems on plasmids. In the first part, we examined the localization of four sRNAs (GlmZ, OxyS, RyhB and SgrS), and the *gfp* mRNA encoding the green fluorescent protein (GFP) using RNA fluorescent *in-situ* hybridization (FISH). We evaluated localization by measuring the overlap of the sRNA signal with the nucleoid or with pixels at the cell membrane. We found that sRNAs localized in both the nucleoid and cytoplasm. In contrast, the *gfp* mRNA control showed less localization in the nucleoid than in the cytoplasm. Further examination of the localization of the sRNAs found that very few cells had membrane localization compared to a control mRNA (*bglF*), which was fused to *gfp* and was known to have membrane localization (29). In the second part of the study, we engineered RNAs, with the *gfp* mRNA as the starting point, to determine whether we could alter nucleoid and cytoplasmic localization. We found that decreasing RNA length and decreasing translation increased nucleoid localization, and that these effects can be combined resulting in the same level of nucleoid localization as the sRNAs. Conversely, we demonstrated that increasing RNA length via fusion of native target mRNA sequences to the *gfp* mRNA, increased the preferential localization of RNAs in the cytoplasm. We also demonstrated that Hfq had no effect on sRNA localization in the cytoplasm and nucleoid. Together our results suggest that RNA size is an important factor, but not the only factor, in determining RNA localization and that because of their small size, sRNAs can enter the nucleoid.

## MATERIALS AND METHODS

### Bacterial plasmids and strains

Strains, plasmids and oligonucleotide sequences are in Supplementary Tables S1, 2 and 3. Plasmids were assembled using components of the pZ system (49) including the ColE1 origin, terminator sequences and promoters. Plasmid maps are in Supplementary Figure S1. sRNA sequences were amplified from MG1655 and cloned downstream of the synthetic PCon (46) promoter (or PConshort promoter for GlmZ (50)). The *gfp* sequence was obtained from pTAK102 (51) and cloned downstream of the PLlacO-1 promoter (49). The *sodB* (−56 to +141) (45), *rpoS* (−149 to +30) and *fhlA* (−107 to +96) mRNA target sequences, and the full length *bglF* sequence (without stop codon) were amplified from MG1655 and translationally fused to the *gfp* mRNA (note: numbering relative to start codon). Chromosomal sRNA genes and *hfq* were deleted using the lambda Red method (52) with the oligonucleotides in Supplementary Table S3.

### RNA fluorescent in situ hybridization (RNA FISH)

RNA FISH was performed on cells inoculated from overnight culture into fresh lysogeny broth (LB) media with 100 μg/ml of ampicillin and grown at 37°C and 200 revolutions per minute (rpm) for 3.5–5 h until they reached an OD_600nm_ ∼ 0.3–0.5, and then they were harvested. Isopropyl β-D-1-thiogalactopyranoside (IPTG; Fisher Scientific, Fair Lawn, NJ, USA) was added as specified for individual experiments in the figure legends and protocols below. The RNA FISH protocol was the same as reported (53) except for the following modifications: (i) the volume of the cell culture and the amount of fluorescent probe were halved; (ii) probes for DsrA, RyhB, SgrS and OxyS sRNAs were in a single mix of DNA probes (labeled with Quasar Cy5); and (iii) GLOX was added after hybridization and washing (final concentration of 0.4% glucose, 10 mM Tris HCL, 2 × SSC, 1% glucose oxidase and 2% of 21.6 mg/ml of catalase from bovine liver) to increase signal and prevent bleaching of Cy3 and Cy5 in accordance with the probes' manufacturer's recommendations (Biosearch Technologies, Novato, CA, USA). Probes were designed using the manufacturer's proprietary software and labeled with Quasar Cy5 for the sRNAs and Quasar Cy3 for the *gfp* mRNA (Supplementary Table S4). Further details are in the Supplementary Data.

Cells were visualized with a Zeiss AxioObserver Z1 inverted microscope with Plan-Neofluar 100×/1.3 oil Ph3 objective and with or without the 1.6× optovar. Images were captured with a Hamamatsu EM-CCD digital camera (Model C9100-13) and iVision-Mac software (Biovision Technologies, Exton, PA, USA). The filter sets are: Cy3 (560/40 nm exciter, 660 nm longpass beamsplitter and 630/75 nm emitter); Cy5 (620/60 nm exciter, 660 nm longpass beamsplitter and 700/75 nm emitter); DAPI (350/50 nm exciter, 400 nm longpass beamsplitter and 420 nm longpass emitter); and GFP (470/40 nm exciter, 495 nm longpass beamsplitter and 525/50 nm emitter). The light source was an X-cite 120Q lamp or X-cite 120LED (Lumen Dynamics, Mississauga, Canada). Power settings, exposure times and gain of the photomultiplier tube detector were adjusted for individual experiments to maximize the signal-to-background ratio.

### Analysis of RNA FISH images

**Figure 1.**
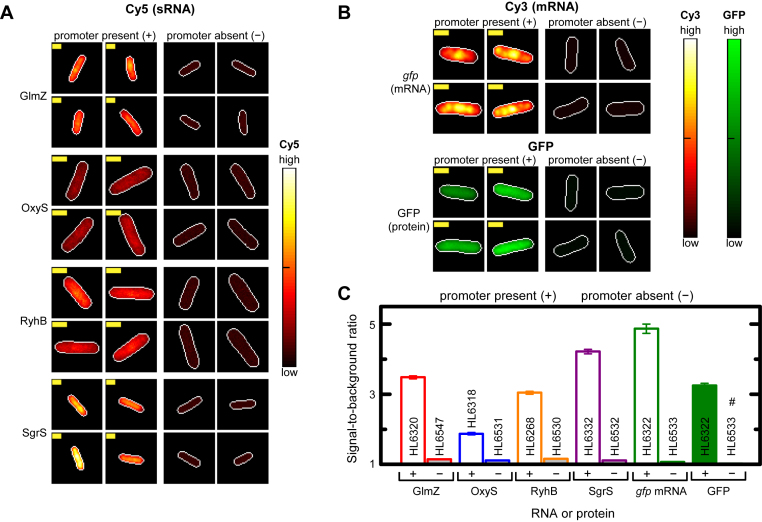
RNA FISH specifically detects sRNAs and mRNAs. (**A** and **B**) sRNA and mRNA signal intensities in representative cells with and without a promoter (PCon or PLlacO-1) in the Cy5 (A), Cy3 (B) or GFP (B) channels. Signal intensity in cells is shown as a heat map with ‘global normalization’ (main text). Cell edges (white line) were identified by phase contrast and transferred to Cy5, Cy3 or GFP channels. Yellow scale bar is 1 μm for all images. Strains with promoter: *glmZ* (HL6320; *n* = 375); *oxyS* (HL6318; *n* = 105); *ryhB* (HL6268; *n* = 233); *sgrS* (HL6332; *n* = 499); *gfp* (HL6322; *n* = 133). Strains with no promoter: *glmZ* (HL6547; *n* = 428); *oxyS* (HL6531; *n* = 281); *ryhB* (HL6530; *n* = 199); *sgrS* (HL6532; *n* = 579); *gfp* (HL6533; *n* = 278). All *gfp* measurements were in the presence of 1 mM IPTG. (**C**) Signal-to-background ratios with and without transcription for each RNA or protein. Error bars are the SEMs. # bar and error are ∼1 and 0 and therefore not visible. Statistical comparison of mean signal-to-background ratios between strains with and without a promoter was significant for all pairs (*P*-values for all pairs < 1 × 10^−58^; Mann–Whitney U two-tailed test and two-tailed *t*-test).

Images were processed in ImageJ (54). The first step (except for the negative control without GFP, Cy3 and Cy5) was alignment of phase-contrast and fluorescence images. This was performed by subtracting background signal (‘Subtract Background’ function), thresholding (default algorithm), aligning thresholded images [customized ‘StackReg’ plugin (55)], extracting offset values from this alignment and applying the offset values to align the original phase-contrast and fluorescence images. Note: background signal is still present in these original images and consequently in the localization analyses. The second step was identification of cells in the phase-contrast images. This was done by thresholding the images (default algorithm) and converting them to binary. On the binary images, cells were initially selected based on size (‘Analyze Particle’ function) and then watershed segmentation (56) was used to separate dividing and touching cells. A second more stringent selection was performed to select cells: (i) with a narrow range of sizes; (ii) that were rod shaped with a major axis to minor axis > 2.01 (AR filter); (iii) that were below a threshold width (MinFeret filter); and (iv) that did not have saturated pixels (Max measurement). Cells with an average signal-to-background < 1.2-fold for Cy3 and < 1.3-fold for Cy5 were not included in the analyses (see Results) except in Figure 1 and for the negative control (HL716). See Supplementary Table S5 for analysis parameters. The cell boundaries were ‘regions of interest (ROI)’. A ‘Count Mask’ was created in ImageJ which filled each ROI within an image with a unique integer. The Count Mask was then used to select pixels in the fluorescence images that correspond to cells using Matlab (R2015a, Mathworks, Natick, MA, USA). Pixel intensity values within each cell were stored in an array with a unique location identifier for each cell.

### Measurements of GFP fluorescence for membrane proteins

Bacteria with BglF::GFP were prepared as follows. An overnight culture was inoculated into fresh LB media with 100 μg/ml of ampicillin and grown at 37°C and 200 revolutions per minute (rpm) for 2–2.75 h to an OD_600nm_ ∼ 0.1. Cells were then induced at 1 mM IPTG for 1 h, grown to an OD_600nm_ ∼ 0.4 and placed on ice for 20 min. One ml of iced culture was centrifuged at 1610 *g*, the supernatant removed and the pellet resuspended in 7 μl of iced LB. Three microliters of resuspended cells were mounted on glass slides with a cover slip. Fluorescence microscopy was performed using a Nikon TE2000 inverted microscope with 100× objective, 1.5 × optovar, with Ph3 annulus, X-cite 120PC lamp (Exfo, Waltham, MA, USA) and an excitation filter/dichroic mirror/emission filter set for GFP (470 ± 20 nm/495 nm/525 ± 25 nm respectively). Images were acquired using a Pixus 1024 pixel CCD camera (Princeton Instruments, Trenton, NJ, USA) and Metamorph 7.0 software (Molecular Devices, Sunnyvale, CA, USA).

### Power calculation for determining selected fraction (F_T_)

To determine the selected fraction of pixels with the highest intensity (F_T_) that were needed to measure overlap with the nucleoid and cell membrane, and thus the threshold, we performed a power calculation assuming: (i) equal numbers of selected pixels for the signal of interest and the center of the nucleoid; (ii) at least 30 cells will be measured and each cell has 300 pixels (i.e. total ‘population’ size = 9000 pixels); (iii) a type I error (α) = 0.05; (iv) power = 1 – type 2 error = 0.8; and (v) the observed overlap of the signal of interest with the center of the nucleoid will be at least 30% of the maximum possible overlap or 30% of the minimum possible overlap (after taking into account the expected overlap of the null distribution which is equal to F_T_). There is no analytical solution so we approximated a function using the Matlab ‘sampsizepwd’ to calculate F_T_ according to the above criteria. We calculated an F_T_ = 0.0886 and rounded to 0.1 for our analyses. Note: with a greater number of cells this selected fraction can detect statistically significant differences in the overlap from the null hypothesis of < 30% of the maximum or minimum possible overlap.

## RESULTS

### sRNAs are in the nucleoid and cytoplasm

We sought to examine the localization of sRNAs in the nucleoid and cytoplasm. The genes for three silencing sRNAs (OxyS, RyhB and SgrS) and two activating sRNAs (DsrA and GlmZ) were placed on plasmids in *E. coli*. The advantages of having the sRNAs on plasmids are: (i) it is more common to synthetic circuits; (ii) it directly examines whether sRNAs can enter the dense structure of the nucleoid (whereas if sRNAs are transcribed from the chromosome it is unclear if their presence in the nucleoid is due to it being their site of production); and (iii) there are multiple copies of the genes which increases the sRNA concentrations thereby making it easier to detect them. Note: one study found no difference in the localization of mRNAs transcribed from a plasmid or from the chromosome (29). We selected the *gfp* mRNA to compare with sRNA localization because it is a non-native mRNA (and therefore less likely to be subject to control mechanisms), a common reporter in synthetic biology and GFP is readily quantified by fluorescence microscopy.

We measured the localization of the sRNAs by RNA FISH because it does not require any modification of the sRNA sequence or structure. Other studies have used RNA FISH to count the number of sRNAs in single cells of *Yersinia pseudotuberculosis* and *Yersinia pestis* (57), to characterize the search kinetics of the SgrS sRNA for *ptsG* (58), and to determine the location of mRNAs (29). Phase contrast microscopy was used to identify the cell boundary and the DNA stain 4΄,6-diamidino-2-phenylindole (DAPI) was used to identify the nucleoid.

In our first experiment we examined whether we could detect sRNAs in exponentially growing cells. We compared sRNA signal intensities between strains with and without transcription of the sRNA (the latter was performed in strains without promoters) using ‘global normalization’ for the RNA signal heat maps (Figure 1A). Global normalization linearly scaled the signals using the highest pixel value in all of the strains (‘high’) and the lowest pixel value in all of the strains (‘low’) to set the range. The normalized signals in each pixel in individual cells were plotted as heat maps. Strong signal was observed with the sRNA probes only in the strains where GlmZ, OxyS, RyhB and SgrS were transcribed and not in control strains without the promoter (Figure 1A and C). These results indicate the probes only detect sRNAs and not their DNA sequences or endogenous RNAs. The signal for the transcribed DsrA was very low therefore no further experiments were performed with it (Supplementary Figure S2). The *gfp* probes were also specific for when the *gfp* mRNA was transcribed (Figure 1B and C).

**Figure 2.**
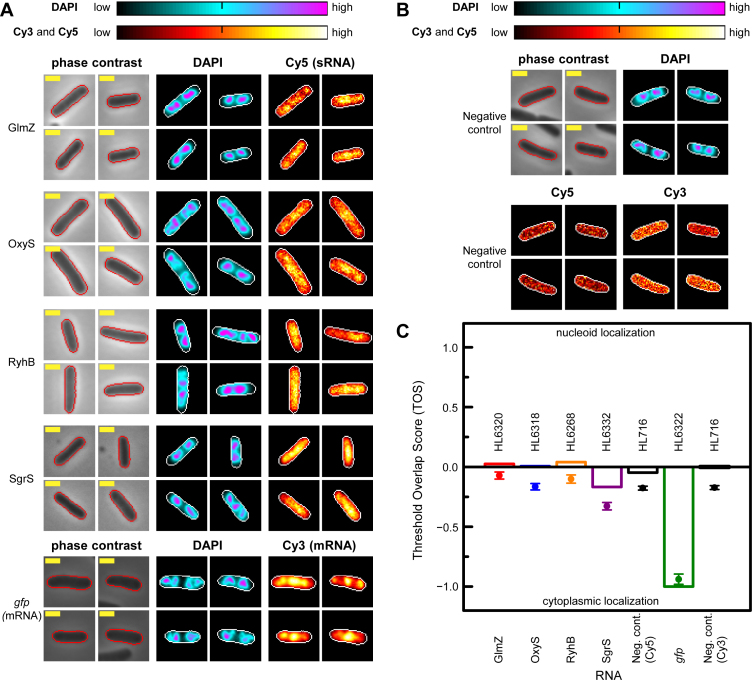
sRNAs occur with equal probability in the nucleoid and cytoplasm. (**A**) sRNA and mRNA localization in representative cells transcribing an sRNA or mRNA in phase contrast, DAPI and Cy5 or Cy3 channels. Signal intensities within cells are shown as heat maps with ‘cellular normalization’ (main text). Cell edges were identified by phase contrast (red line) and transferred to the DAPI, Cy5 and/or Cy3 channels (white line) following alignment. Yellow scale bar indicates 1 μm. Strains with promoter: *glmZ* (HL6320; *n* = 81); *oxyS* (HL6318; *n* = 126); *ryhB* (HL6268; *n* = 93); *sgrS* (HL6332; *n* = 209); *gfp* (HL6322; *n* = 34). Data for *gfp* are from the same experiment as in Figure 1 but here they are shown with cellular normalization instead of global normalization (note: cells with saturated pixels in the DAPI channel were not included in this analysis). (**B**) Representative cells in the negative control (HL716; *n* = 355) without Cy3 or Cy5 probes in phase contrast, DAPI, Cy5 and Cy3 channels. The negative control is the host strain without any plasmid (HL716) and without probes. (**C**) Threshold overlap score (TOS) for each sRNA, the mRNA and negative control. Bars are the medians, circle symbols are the means and error bars are the SEMs. TOS is a normalized measure of the overlap of the top 10% of the DAPI and Cy5 (or Cy3) signals (see main text for further details). Median TOS of each sRNA to the *gfp* mRNA was significantly different (*P* = 2.0 × 10^−14^, 6.0 × 10^−16^; 9.0 × 10^−15^ and 2.5 × 10^−11^ for GlmZ, OxyS, RyhB and SgrS respectively; Mann–Whitney U two-tailed test).

To visualize intracellular localization of sRNAs and mRNAs in individual cells we performed ‘cellular normalization’ for the RNA signal heat maps rather than ‘global normalization’. That is, we linearly scaled the pixel values in each cell using the highest (‘high’) and lowest (‘low’) pixel value for that particular cell (Figure 2A). Inspection of representative cells indicated that pixels with the highest intensity signal for each sRNA appear to occur in regions with DAPI (i.e. in the nucleoid) as well as regions outside it (i.e. in the cytoplasm). The *gfp* mRNA displayed a very different pattern of localization with high signal predominantly in the cytoplasm (Figure 2A). The negative control strains for Cy3 and Cy5 (described in figure legend) had diffuse localization of signal as expected for background signal (Figure 2B). The signal-to-background ratio was <1.2 and <1.3 for Cy3 and Cy5 respectively in > 95% of the negative control cells; these values were used as cut-offs to identify cells in our experiments with no signal and therefore not included in the analyses.

The nucleoid does not have a distinct boundary but instead has parts that extend into the cytoplasm. Therefore to evaluate whether sRNAs can enter the nucleoid we need to focus on their localization in the center of the nucleoid where the DNA is densest and the DAPI signal is highest. Specifically, we needed to set a threshold to select the pixels with the highest DAPI signal. However, we did not want to set the threshold too high so that there were too few pixels to evaluate the statistical significance of the measured overlap of the sRNA signal with the center of the nucleoid. In other words, the threshold needs to select a fraction of pixels with the highest intensity signal (F_T_) that is neither too small nor too large. We performed a power calculation (‘Materials and Methods’ section) and determined F_T_ = 0.1 to be the threshold for our experiments. That is, we set thresholds for the sRNA and DAPI signals (and also for the mRNA and GFP signals) so that the 10% of pixels with the highest intensities in each cell were selected for our analyses.

To quantify localization we first determined the observed overlap of our selected pixels for the sRNA signal (or *gfp* mRNA signal) with our selected pixels for the DAPI signal (i.e. the center of the nucleoid). This was calculated by counting the number of overlapping pixels and dividing it by the number of selected pixels for the DAPI channel. Because we selected the pixels with the highest signal, the effect of background signal was minimal. We then divided the observed overlap by the expected overlap for a uniform distribution of random signal intensities across the cell. We rescaled this ratio resulting in a threshold overlap score (TOS) with −1 and +1 as the minimum and maximum respectively (59). That is,

\begin{eqnarray*} &&{\rm{TOS}} = \nonumber \\ &&\left\{ {\begin{array}{@{}*{1}{l}@{}} {0,\ \,{\rm{when}}\,{\rm{observed}}\,{\rm{overlap}}\ = \ {\rm{expected}}\,{\rm{overlap,}}}\\ {\frac{{{\rm{observed\ }}\,{\rm{overlap}}}}{{{\rm{expected}}\,{\rm{overlap}}}}{\rm{\ - 1,\ }}\,{\rm{when}}\,{\rm{observed\ }}\,{\rm{overlap\ < \ expected}}\,{\rm{overlap,}}\,{\rm{and}}}\\ {\left( {\frac{{{\rm{observed}}\,{\rm{overlap}}}}{{{\rm{expected}}\,{\rm{overlap}}}}{\rm{ - 1}}} \right){{\left( {\frac{{\rm{1}}}{{{\rm{expected}}\,{\rm{overlap}}}}{\rm{ - 1}}} \right)}^{{\rm{ - 1}}}}{\rm{,}}\,{\rm{when}}\,{\rm{observed}}\,{\rm{overlap\ >\ expected}}\,{\rm{overlap}}{\rm{.}}} \end{array}} \right. \end{eqnarray*}

The observed and expected overlaps are both fractions. The expected overlap is 0.1. TOS > 0, ≈ 0 and < 0 indicate the sRNA or mRNA occurs in the nucleoid more, the same, or less than a signal that is uniformly distributed throughout the cell (i.e. colocalization, non-colocalization and anticolocalization with the nucleoid respectively). It is important to note that TOS is designed to evaluate the fractional overlap of the sRNA signal or mRNA signal with DAPI independent of the level of clustering of selected pixels (see summary below). Otherwise, any change in localization measured by the TOS metric would be due to an unknown combination of changes in signal overlap and/or clustering.

Our analysis revealed that three sRNAs (GlmZ, OxyS and RyhB) had median TOS values of 0.02, 0.01 and 0.04 respectively (Figure 2C). That is, the top 10% of pixels with the highest intensity signal for each sRNA and the ‘center’ of the nucleoid (where the top 10% of pixels with the highest intensity signal for DAPI occur) have TOS values that are close to zero. This indicates the sRNA signals overlap as much as would be expected for a signal that was uniformly distributed in the cell (i.e. non-colocalization). For SgrS, there was weak anticolocalization of the signal and the nucleoid (median TOS ≈ −0.17). In contrast to the sRNAs, *gfp* mRNA has strong anticolocalization with a median TOS = −1.00 (Figure 2C). Therefore there is essentially no overlap between the top 10% of pixels with highest intensity *gfp* mRNA signal and the top 10% of pixels with highest intensity DAPI signal. To better highlight this difference between sRNAs and mRNA localization we show only the 10% of pixels with highest signal intensity for the sRNA or mRNA (magenta color), and only the 10% of pixels with highest signal intensity for the DNA (i.e. DAPI; cyan color) in Supplementary Figure S3. Selected pixels for the sRNAs or mRNAs that overlap the selected pixels for the DNA have a yellow color. Supplementary Figure S3 shows that relatively few of the selected pixels for the mRNAs overlap the selected pixels for the DNA compared to the sRNAs (note: this can be most clearly seen in ‘zoomed’ views of the digital images).

In summary, sRNAs tend to occur in both the nucleoid and cytoplasm and the *gfp* mRNA occurs predominantly in the cytoplasm. The mechanistic basis for this difference is examined in later experiments. Our finding that the fractional overlap of the 10% of pixels with highest intensity signals for the sRNAs and the 10% of pixels with the highest intensity signal for the DNA, is approximately the same as expected by chance for a uniform distribution, does not necessarily mean the pixels for *both* signals are actually uniformly distributed throughout the cell. First, it is sufficient for the pixels of only one of the signals to be uniformly distributed. Second, TOS evaluates overlap independent of the level of clustering of the selected pixels. In the second case, the selected pixels for the RNA signal and the DAPI signal may be clustered but if these clusters are randomly distributed in the cell then the overlap may be same as expected for a uniformly distributed signal.

### sRNAs display no preferential membrane localization

We analyzed images from the above experiments to determine if there was increased localization of sRNAs at the cell membrane. We included the *gfp* mRNA as a negative control because it does not localize at the membrane (30,60). In addition, we created a positive control by fusing the *bglF* mRNA, which encodes β-glucoside phosphotransferase permease (BglF), to the *gfp* mRNA. BglF has eight domains that span the inner membrane (61) and the *bglF* mRNA has previously been shown to localize to the membrane by a translation independent mechanism (29). RNA FISH was performed as above and GFP was measured by fluorescence microscopy (‘Materials and Methods’ section).

**Figure 3.**
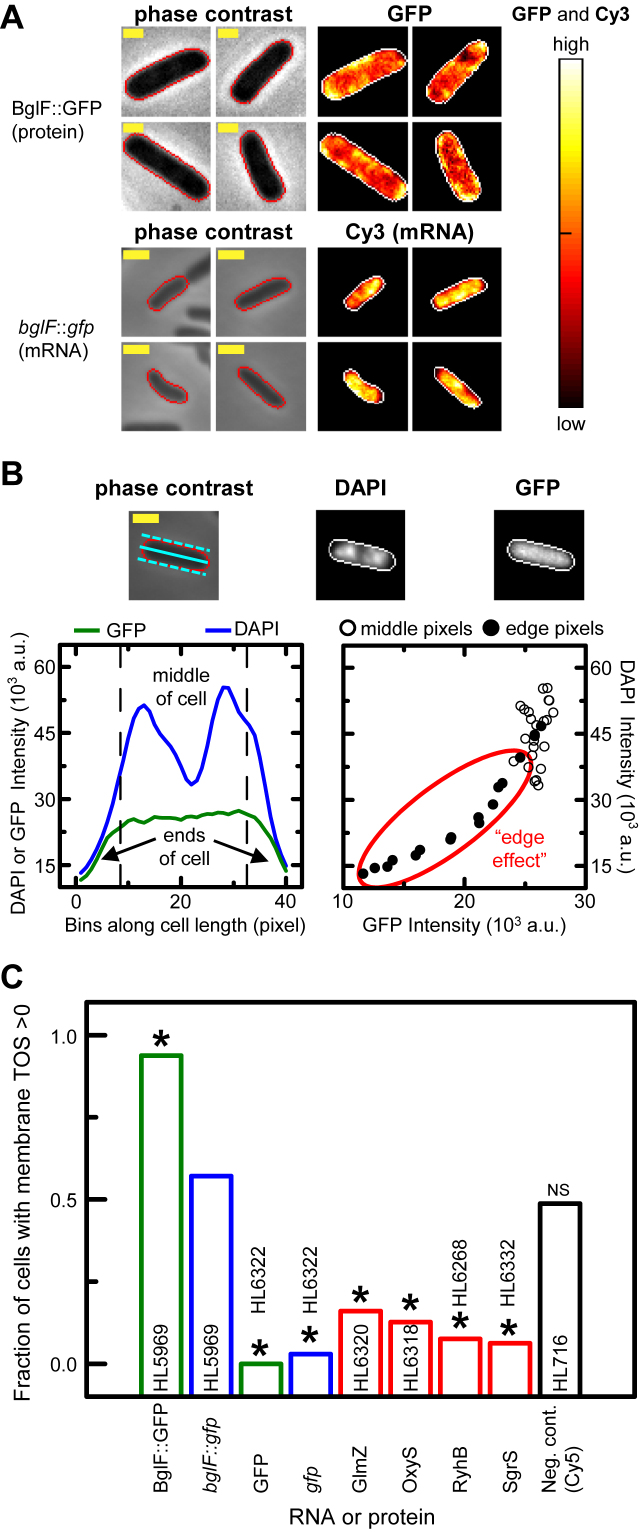
sRNAs display no preferential membrane localization. Cell edges were identified as in Figure 2A. Yellow scale bar indicates 1 μm. (**A**) mRNA and protein localization of *bglF*::*gfp* (HL5969) in representative cells shown for the phase contrast, GFP (*n* = 304) or Cy3 (*n* = 84) channels. Signal intensities of Cy3 and GFP are shown as heat maps with cellular normalization. Measurements were made at 1 mM IPTG. (**B**) ‘Edge effect’ shown in a cell with GFP expression (HL6322). Average GFP fluorescence and DAPI signals along the longitudinal axis (upper cell images); both signals decrease at the cell ends (left lower plot) resulting in a positive correlation for binned pixel values (right lower plot; circled with a red line). Solid and dash cyan lines in the phase contrast image show the center and edges of the longitudinal axis. (**C**) Fraction of cells with membrane localization (‘membrane TOS’ > 0) in strains probed for sRNAs and mRNAs, or expressing GFP. Analysis was performed on measurements collected for Figure 2 (HL6322, HL6320, HL6318, HL6268, HL6332, and HL716). The number of cells with TOS > 0 was compared between the positive control (*bglF*::*gfp*) and each of the sRNAs and mRNAs using Fisher's exact test. * indicates statistical significance (*P* < 1 × 10^−6^). NS indicates no significance (*P* = 1.8 × 10^−1^) for the negative control (Cy5).

The BglF::GFP protein was observed in regions near the membrane as expected (Figure 3A). The *bglF*::*gfp* mRNA was not obviously at the membrane from visual inspection of cell images but it was detectable by quantitative analysis. Quantitative analysis was performed by identifying the cell boundaries in the phase contrast images and removing the outermost layer of pixels using the ‘erode’ function in ImageJ. The outermost layer of pixels were removed because they include areas outside the cell and have less signal from the point spread function of neighboring pixels. Together these factors create an ‘edge effect’ with lower signal in the outermost layer of pixels (Figure 3B). The next outermost layer was termed the ‘membrane’ and we determined by TOS whether the top 10% of intensity values in the whole cell overlap with this membrane layer more, less or the same as a uniform distribution. We created histograms of this ‘membrane TOS’ obtained from each cell and found that the mean and median values often did not capture differences between samples because of heterogeneity in the cell populations (Supplementary Figure S4). Therefore we measured the fraction of cells in each sample that had colocalization of the sRNA or mRNA signal with the membrane region (i.e. membrane TOS > 0) (Figure 3C).

BglF::GFP and *bglF*::*gfp* mRNA had 93.8 and 57.1% of cells with membrane TOS > 0 respectively (Figure 3C). That is, the 10% of pixels with the highest BglF::GFP and *bglF*::*gfp* mRNA signal in the cell overlapped with the membrane region more than expected by random chance in the majority of cells. These results in the positive controls are consistent with membrane localization. In contrast, all the sRNAs and the *gfp* mRNA had very low percentages of cells with TOS > 0 (Figure 3C), and these percentages were significantly lower than the *bglF::gfp* mRNA positive control (*P*-values < 1 × 10^−6^; Fisher's exact test). The control with uniform randomly distributed background signal (HL716) is not affected by the edge effect and was expected to have a 50:50 random split of cells with TOS > 0, and this was observed (Figure 3C). In summary, none of the sRNAs displayed evidence of membrane localization.

### RNA length and translation affect nucleoid localization

We attempted to engineer RNAs with the same level of nucleoid localization as sRNAs. Creating increased nucleoid localization for the full length *gfp* mRNA was judged to be more likely to be informative than simply disrupting nucleoid localization of sRNAs. Moreover, sRNAs have very important structure-function relationships that when altered could have unexpected and unexplainable effects. The first factor that was considered was RNA length. RyhB, OxyS, GlmZ and SgrS have lengths of 102, 121, 207 and 238 nucleotides respectively whereas the *gfp* mRNA has a total length of 994 nucleotides (717 nucleotides for the coding sequence and ∼277 nucleotides for the 5΄ untranslated region and T1 terminator). Therefore the *gfp* mRNA is expected to have a larger size resulting in more difficulty diffusing through the compact chromosomal DNA of the nucleoid. The second factor that was considered were polysomes, which are complexes comprised of an mRNA, 70S ribosomes, the translated peptide and other factors (62). The presence of polysomes increases mRNA size and therefore could impede mRNA movement through the nucleoid.

**Figure 4.**
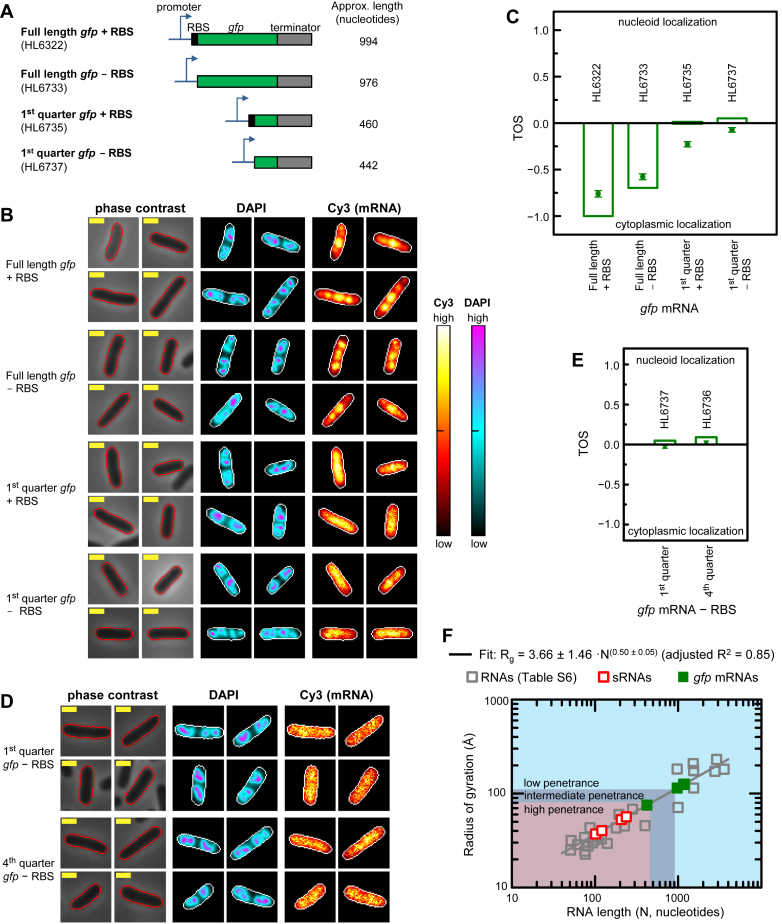
RNA length and translation affect nucleoid localization. Cell edges were identified as in Figure 2A. Yellow scale bar indicates 1 μm for all images. Measurements were made at 1 mM IPTG. (**A**) Full and partial length *gfp* mRNA with and without the RBS (st7) and start codon. (**B**) Localization of *gfp* mRNA in representative cells with each of the genes in panel A. DAPI and Cy3 signal intensities (cellular normalization) represented as heat maps. Sample sizes: HL6322 (*n* = 113), HL6733 (*n* = 186), HL6735 (*n* = 229) and HL6737 (*n* = 209). (**C**) TOS for strains with each of the genes shown in panel A. Bars are the medians, circle symbols are the means and error bars are the SEMs. Median TOS for the following pairwise combinations were statistically significant (Mann–Whitney U two-tailed test): full length *gfp* mRNA ± RBS (*P* = 2.6 × 10^−4^), first quarter *gfp* mRNA ± RBS (*P* = 3.8 × 10^−4^) and full length *gfp* mRNA – RBS versus first quarter *gfp* mRNA – RBS (*P* = 1.4 × 10^−27^). (**D**) Comparison of *gfp* mRNA localization in cells with first quarter *gfp* mRNA – RBS (HL6737; *n* = 231) and fourth quarter *gfp* mRNA – RBS (HL6736; *n* = 205). (**E**) TOS for strains with the genes in panel D. Plot is presented as in panel C. The difference in median TOS was small and barely significant (*P* = 1.2 × 10^−2^, Mann–Whitney U two-tailed test). (**F**) Radius of gyration (R_g_) as a function of RNA length. R_g_ values from the literature, which are provided in Supplementary Table S6, were fitted to a power function as defined in main text (gray line). Parameter values from the fit were then used to calculate R_g_ for the sRNA and *gfp* mRNAs without RBS. Two target mRNA::gfp mRNA fusions from Figure 5 are included in the plot for comparison. Because these fusions (*rpoS::gfp* and *fhlA::gfp*) have similar lengths their symbols overlap (see Figure 5 and main text for more details). Parameter errors are the standard deviations. Shading shows RNA size ranges that may have potentially high, intermediate and low nucleoid penetrance.

To evaluate the effects of RNA length and polysome formation on localization we compared the full length *gfp* mRNA to a partial length mRNA, and both lengths with and without translation. Translation was prevented by deleting the ribosome binding sequence (RBS) and the start codon, which for the full length mRNA abolished GFP fluorescence. To summarize, there were four sets of samples in this experiment (Figure 4A): (i) full length *gfp* mRNA with translation (≈994 nucleotides); (ii) full length *gfp* mRNA without translation (≈976 nucleotides); (iii) first quarter of the *gfp* mRNA with an introduced stop codon and translation (≈460 nucleotides); and (iv) first quarter of the *gfp* mRNA with an introduced stop codon and no translation (≈442 nucleotides). Localization of these mRNAs was measured by RNA FISH using the Cy3 probes for the *gfp* sequence (representative cells in Figure 4B). TOS was calculated as described above to measure the overlap of pixels with the top 10% of the mRNA signal and pixels with the top 10% of the DAPI signal (which are primarily in the center of the nucleoid). We found that in most samples, the median and mean TOS were not the same due to a long-tailed distribution. This distribution required a rank-order test for statistical significance (Mann–Whitney U two-tailed test) and therefore we primarily compared the median TOS between samples.

We examined the effect of translation on localization by comparing median TOS for full length mRNA with and without the RBS (−1.00 and −0.70 respectively) (Figure 4C). These differences were statistically significant (*P* = 2.6 × 10^−4^; total *n* = 299). Therefore decreasing translation to reduce the number of polysomes along the mRNA increased the overlap of pixels with the highest (i.e. top 10%) *gfp* mRNA and DAPI signals; that is, polysomes appear to prevent nucleoid localization of the mRNA. We next examined the effect of mRNA length on localization by comparing median TOS for the full length *gfp* without the RBS and the first quarter of *gfp* without the RBS, which were −0.70 and 0.05 respectively (Figure 4C). The difference in median TOS was statistically significant (*P* = 1.4 × 10^−27^; total *n* = 395) indicating that decreasing mRNA length enabled greater localization of the top 10% of pixels with the *gfp* mRNA signal in the nucleoid. We did not compare full length *gfp* with the RBS to the quarter length *gfp* with the RBS because altering mRNA length can also potentially decrease the number of polysomes along the mRNA; therefore observed differences may reflect the combined effects of length and translation. It is notable that the partial length *gfp* mRNA without an RBS has a median and mean TOS ≈ 0; that is, it has the same fractional overlap with the nucleoid as the sRNAs.

The partial length *gfp* mRNAs with and without an RBS had similar median TOS (0.01 and 0.05 respectively) but large differences in mean TOS (−0.23 ± 0.03 and −0.07 ± 0.02 respectively) (Figure 4C). This difference in the means was due to the *gfp* mRNA without the RBS having more cells in the tail of the distribution with greater localization of mRNA in the cytoplasm. The data indicate the effects of shortening RNA length and decreasing translation can be combined, which is expected if polysomes and length contribute at least partly independently to RNA size, and if RNA size affects nucleoid localization.

We replaced the first quarter of the *gfp* coding sequence without the RBS and start codon with the sequence from the last quarter of the *gfp* coding sequence and repeated the experiments and analysis (representative cells in Figure 4D). Despite the different sequences, these partial length *gfp* mRNAs had very similar median and mean TOS (Figure 4E), which suggests the degree of nucleoid localization is primarily determined by length and not sequence.

To determine how RNA lengths relate to their size, we plotted the radius of gyration (R_g_) for sRNAs, ribozymes, transfer RNAs, ribosomal RNAs and mRNAs (Figure 4F and Supplementary Table S6). The radius of gyration is a way of describing the distribution of mass of an RNA or protein around its axes of rotation. If the shape of a RNA or protein is approximated by a solid sphere then the diameter is roughly equal to the radius of gyration multiplied by 2√(5/3) (63). The values were obtained from the literature or by searching the Nucleic Acid Database Project for bacterial RNA structures with >30 nucleotides and without any protein binding (64). We fitted the measurements to the power law relationship that exists between R_g_ and the number of bond segments for a polymer. Specifically, we used the function R_g_ = a·N*^ν^*, where N is the number of nucleotides, *a* is a pre-factor and *v* is an exponent that specifies the compactness of the RNA in a solvent (65–69).

Our fit using the Levenberg-Marquardt algorithm yielded *v* = 0.50 ± 0.05 and *a* = 3.66 ± 1.46 Å (adjusted *R*^2^ = 0.85; *n* = 28). The exponent (*v*) is consistent with an ideal polymer chain with a simple random walk in a θ solvent (65). Studies of relatively short RNAs, which tend to be tRNAs, riboswitches and ribozymes with high levels of self-annealing and more compact structures, often have exponents ≈1/3 to ≈2/5 (67,70). We reanalyzed the data for only the tRNAs, riboswitches and ribozymes (51–400 nucleotides), and the fit yielded an exponent *v* = 0.36 ± 0.10 (*a* = 6.18 ± 3.25 Å; adjusted *R*^2^ = 0.53; *n* = 13), which was similar to that reported in the other studies (67,70). Conversely, our fit to experimentally measured sRNAs, mRNAs and random sequences (75–1523 nucleotides) yielded a high exponent value indicating self-avoiding interactions and a larger volume (*v* = 0.60 ± 0.11 and *a* = 2.68 ± 2.04 Å; adjusted *R*^2^ = 0.98; *n* = 5). Note: all errors for the fits are the standard deviations.

We estimated the R_g_ for our sRNAs using the parameters from the first fit to all the RNAs because it had the lowest relative errors and was the most general fit. The calculated R_g_ for the sRNAs are: 37.0 Å (RyhB, 102 nucleotides); 40.3 Å (OxyS, 121 nucleotides), 52.7 Å (GlmZ, 207 nucleotides) and 56.5 Å (SgrS, 238 nucleotides). The predicted R_g_ for the partial length *gfp* mRNA without the RBS is 75.2 Å. This value is only slightly larger than for the sRNAs and similar to the 30S and 50S ribosomes which are ∼70–80 Å (71–73) and can enter the nucleoid (37).

The predicted R_g_ for the full length *gfp* mRNA without the RBS is 114.3 Å, which is approximately the same radius as the 70S ribosome (74,75). That is, the R_g_ for the full length *gfp* mRNA, which does not enter the nucleoid, is 1.5-fold larger than the R_g_ of the partial length *gfp* mRNA that does enter the nucleoid. While this fold difference is relatively small, the absolute difference in diameter is large (≈100 Å) (note: diameters of the partial and full length *gfp* mRNAs are 194.1 Å and 295.2 Å respectively, assuming they are spherical).

**Figure 5.**
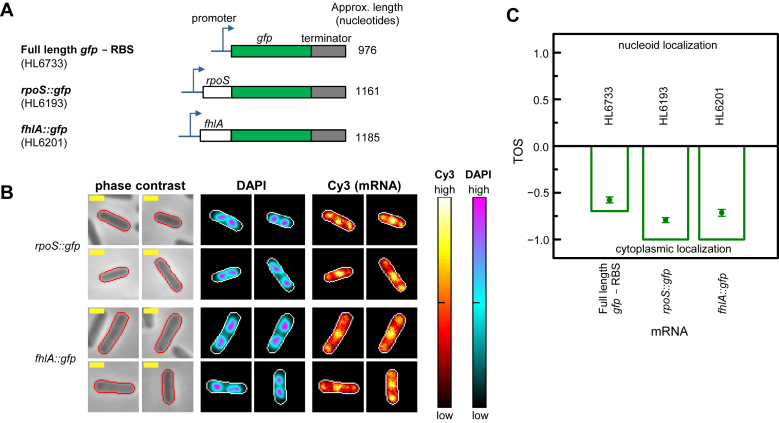
Localization of target mRNAs fused to *gfp*. Cell edges were identified as in Figure 2A. Yellow scale bar indicates 1 μm for all images. Measurements were made at 1 mM IPTG. Note: HL6733 data are the same as that shown in Figure 4 and are reshown to enable convenient comparison. (**A**) Full length *gfp* mRNA with and without additional untranslated and coding sequences. (**B**) Localization of *gfp* mRNA in representative cells with each of the genes in panel A. DAPI and Cy3 signal intensities (cellular normalization) represented as heat maps. Sample sizes: HL6733 (*n* = 186), HL6193 (*n* = 177) and HL6201 (*n* = 98). (**C**) TOS for strains with each of the genes shown in panel A. Bars are the medians, circle symbols are the means and error bars are the SEMs. Differences in the TOS values for the following pairwise combinations of samples were statistically significant (Mann–Whitney U two-tailed test): full length *gfp* mRNA – RBS versus *rpoS::gfp* mRNA (*P* = 2.3 × 10^−7^) and full length *gfp* mRNA – RBS versus *fhlA::gfp* mRNA (*P* = 1.2 × 10^−2^).

We also measured the localization of mRNAs that are longer than the full length *gfp* mRNA. These mRNAs had the non-translated region and partial coding sequence of two native mRNAs, *rpoS* and *fhlA*, translationally fused to *gfp* (Figure 5A). These native mRNAs are known targets for sRNA regulation, and both fusions were previously described and shown to have relatively low translation, particularly for *fhlA::gfp* (46). Because of this low level of translation, it was more appropriate to compare the localization of these target mRNA::*gfp* fusions to the localization of the full length, non-fusion *gfp* mRNA without the RBS rather than with the RBS. The lengths of the *rpoS::gfp* and *fhlA::gfp* fusion mRNAs were ≈1161 and ≈1185 nucleotides respectively. We measured localization by the same method as used for the other *gfp* mRNAs, and determined the mean TOS to be −0.79 ± 0.03 and −0.71 ± 0.04 for *rpoS::gfp* and *fhlA::gfp* respectively, and the median TOS to be −1.00 for both mRNAs (Figure 5B and C). The *rpoS::gfp* and *fhlA::gfp* fusion mRNAs, which are longer than the full length *gfp* mRNAs and have even less overlap with the nucleoid, have predicted R_g_ of 124.7 Å and 126.0 Å respectively, and diameters of 322.0 Å and 325.3 Å respectively (Figure 4F). These results show that mRNAs longer than the full length *gfp* mRNA without the RBS have even greater localization in the cytoplasm, consistent with their longer length further reducing nucleoid localization.

Together the data indicate that RNAs can be engineered to increase or decrease their nucleoid localization by altering their length and/or level of translation to increase or decrease their size. For RNAs to have the same level of nucleoid localization as sRNAs, they appear to require an R_g_ < ≈ 80 Å or diameter < ≈ 200 Å, and no translation, with all other factors being equal. Larger RNAs appear to have more difficulty moving into and through the nucleoid, and thus tend to localize outside the nucleoid.

### Hfq has minimal effect on sRNA localization

Given the prominent role of Hfq in regulating sRNA activity in *E. coli* it is important to establish whether Hfq affects sRNA localization. Hfq could potentially affect sRNA localization in several ways. The Hfq hexamer has a diameter of 62–65 Å (76,77) therefore its binding to sRNAs could potentially add to their size and limit their movement in nucleoid regions with the densest DNA. Alternatively, the binding of Hfq to sRNAs may decrease their size as occurs with the *rpoS* target mRNA, which has a smaller size in the Hfq::*rpoS* complex (R_g_ = 58.0 ± 1.0 Å) than alone (R_g_ = 68.1 ± 1.6 Å) (78). In addition, Hfq can bind to DNA (79) therefore it could potentially sequester sRNAs in the nucleoid.

**Figure 6.**
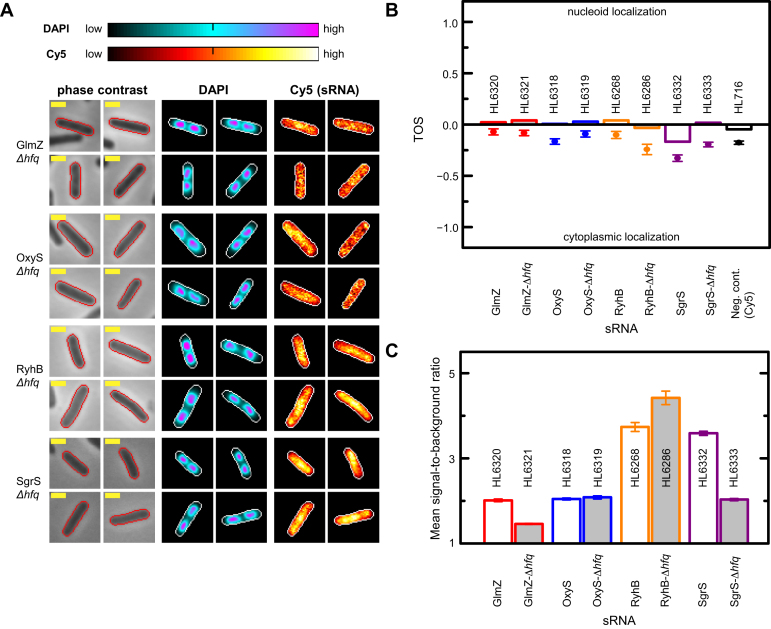
Hfq has minimal effect on sRNA localization. Cell edges were identified as in Figure 2A. Yellow scale bar indicates 1 μm for all images. Measurements for the negative control strain (HL716) without any plasmid and probes were made at 1 mM IPTG. (**A**) sRNA localization in strains without *hfq* (Δ*hfq*). Signal intensities of DAPI and Cy5 in individual cells are shown as heat maps with cellular normalization. All strains have the sRNA transcribed from the PCon promoter. Strains: *glmZ* (HL6321; *n* = 144); *oxyS* (HL6319; *n* = 97); *ryhB* (HL6286; *n* = 62); and *sgrS* (HL6333; *n* = 326). (**B**) TOS for each sRNA with *hfq* (data from the experiments in Figure 2A) and without *hfq* (panel A). Bars are the medians, circle symbols are the means and error bars are the SEMs. Median TOS for pairs of strains with or without *hfq* were very similar for GlmZ, OxyS, and RyhB (*P* = 4.4 × 10^−1^, 1.3 × 10^−2^ and 1.7 × 10^−2^ respectively; Mann–Whitney U two-tailed test). Median TOS was significantly different for SgrS with or without *hfq* (*P* = 5.2 × 10^−4^). (**C**) Signal-to-background ratios for each sRNA with and without *hfq*. Error bars are the SEMs. Mean signal-to-background ratio in strains with and without *hfq* were statistically significant for GlmZ, RyhB and SgrS but not for OxyS (*P* = 1.4 × 10^−50^, 2.6 × 10^−4^, 4.0 × 10^−125^ and 3.7 × 10^−1^ respectively; two-tailed *t*-test).

We examined sRNA localization in strains without Hfq (Δ*hfq*) by RNA FISH (Figure 6A). These measurements were performed in parallel in strains with Hfq (Figure 2). We calculated TOS and found little or no difference in nucleoid localization with and without Hfq (Figure 6B). The exception was SgrS, which lost its slight preference for the cytoplasm with the deletion of *hfq* resulting in equal preference for the cytoplasm and the nucleoid (median TOS = −0.17 and 0.02 respectively; *P* = 5.23 × 10^−4^; total *n* = 535). The deletion of *hfq* prevents SgrS from forming duplexes with its target mRNA (*ptsG*) (80), therefore the latter finding of decreased anticolocalization with the nucleoid is probably due to less SgrS binding to its target mRNA outside the nucleoid at the cell poles (58). It should be noted that under our growth conditions it has been established that SgrS translation does not occur (80,81).

The minimal effects of Hfq on sRNA localization are unlikely to be due to the sRNAs being in such excess that there is insufficient Hfq to bind most of the sRNA molecules. One reason it is unlikely that there is a large pool of unbound sRNAs, is that many studies have shown that sRNAs are rapidly degraded in the absence of Hfq [see references and data in (42,46)]. Furthermore, Hfq is clearly interacting with at least GlmZ, RyhB and SgrS because the deletion of *hfq* altered their mean signals (and thus their concentrations) (Figure 6C). The halving of the GlmZ and SgrS concentrations (mean signal above background) when *hfq* was deleted suggests that at least 50% of the GlmZ and SgrS sRNAs are bound to Hfq (see calculations and model in the Supplementary Data). If Hfq was only binding to a small fraction of these sRNAs then the deletion of *hfq* should have had a correspondingly small effect on their concentrations.

There are many reasons OxyS and RyhB may have shown no difference in concentration and an increase in concentration with the deletion of *hfq*. These include similar degradation rates for the unbound and Hfq bound forms in our experiments, and decreased duplex formation in the *hfq* deletion strains causing increases in the sRNA concentrations by amounts that offset (OxyS), or more than offset (RyhB), the decreases in sRNA concentrations caused by the loss of Hfq protection of the sRNAs. We tested duplex formation of RyhB with a fusion of its target sequence (*sodB*) to *gfp* and found that SodB::GFP levels were increased in the Δ*hfq* strain (signal-to-background ratio in wild-type and Δ*hfq* strains were 3.28 ± 0.18 and 6.54 ± 0.46; HL6284 and HL6285). Therefore RyhB activity was decreased in the Δ*hfq* strain, and consequently Hfq concentrations in the wild-type background are sufficiently high to be a major contributor to duplex formation.

Together our experiments indicate the similarity in sRNA localization in the wild-type and Δ*hfq* strains is not explained by insufficient Hfq, and is most likely due to Hfq not having much effect on sRNA localization.

## DISCUSSION

In this study we showed that sRNAs occur throughout the nucleoid and cytoplasm. The four sRNAs that were measured are diverse: (i) they ranged in size from 102 to 238 nucleotides; (ii) one (GlmZ) acts to increase target protein production and three (OxyS, RyhB and SgrS) act to decrease target protein production; and (iii) they are involved in regulating different classes of proteins in different pathways including iron storage (RyhB), oxidative stress (OxyS) and carbohydrate metabolism (GlmZ and SgrS) (1). Given the variety of the sRNAs studied, the findings of this study are likely to be general. In contrast to the sRNAs, the full length *gfp* mRNA almost exclusively occurs in the cytoplasm, which is consistent with nucleoid exclusion and the observation in another study that diffusion of *gfp* mRNA appeared to avoid the nucleoid (30). We hypothesized that the difference between the sRNAs and the *gfp* mRNA was due to the larger size of the latter because of its greater length and polysomes. Decreasing each of these factors reduced nucleoid exclusion of the full length *gfp* mRNA, and decreasing both of these factors completely eliminated the nucleoid exclusion. Together the results indicate that sRNAs are able to enter the nucleoid due to their smaller size, and our observation that there is no preferential localization in the cytoplasm or nucleoid suggests that sRNAs probably move into and out of the nucleoid at similar rates (see below).

We also found that the deletion of *hfq* had minimal effect on the localization of sRNAs. This result includes sRNAs that bound to Hfq in our experimental systems in sufficient amounts that the deletion of *hfq* affected their concentrations and/or activities. To explain our findings we consider three plausible scenarios that take into account Hfq stabilizes sRNAs (1,11,46). In scenario 1, Hfq binds sRNAs in the nucleoid or the cytoplasm and unbound sRNA movement into and out of the nucleoid is slow or limited. In this scenario we would expect the concentration to decrease at the site of Hfq binding resulting in decreased or increased nucleoid localization, which is not consistent with our observations. In scenario 2, Hfq binds sRNAs in the nucleoid or the cytoplasm, and unbound sRNA movement into and out of the nucleoid is fast and unlimited. In this scenario we would expect the deletion of *hfq* to decrease the total cellular sRNA concentration but there would be minimal effect on localization because of rapid movement of sRNAs. In scenario 3, Hfq binds sRNAs in both the cytoplasm and nucleoid. For the sRNAs to localize with equal probability in the nucleoid and cytoplasm as observed, Hfq must also localize with equal probability in the nucleoid and cytoplasm (or less likely, there is a difference in Hfq activity in the nucleoid and cytoplasm that is exactly counterbalanced by a difference in Hfq concentration in the nucleoid and cytoplasm so the sRNA localization appears to occur with equal probability in the nucleoid and cytoplasm). In this scenario, deletion of *hfq* decreases the concentration of sRNAs in both the nucleoid and the cytoplasm. However, for sRNA localization not to change with the deletion of *hfq* (as was observed), then the sRNAs must also be able to move without Hfq equally to the nucleoid and cytoplasm. Scenarios 2 and 3 are compatible with our observations, and both are consistent with sRNAs being able to move in and out of the nucleoid with minimal bias. To be clear, our findings do not specify where in the cell sRNAs bind and act on mRNAs.

Our observation that sRNAs can readily move into and through the nucleoid indicates they have the potential to bind mRNAs at the earliest stages of transcription and therefore can compete with ribosomal subunits for binding at the TIR. As mentioned above, the advantage of sRNAs binding to the TIR before the ribosomal subunits (instead of waiting until afterward), is that it can potentially prevent the first round of translation initiated within the nucleoid and therefore prevent any protein at all being produced. This advantage is important for target proteins that exert their actions at low concentrations (82) and in systems that have high cooperativity, ultrasensitive switches or positive feedback (83,84). As an example, there is an advantage to blocking the first round of translation to completely inhibit the production of some outer membrane proteins (such as OmpA, OmpC and OmpF, which are regulated by the MicA, MicC and MicF sRNAs), for which the expression of even a single protein could provide a route for bacteriophage to enter the cell and cause cell death (85). In addition, if sRNAs bind to the TIR immediately after its transcription they can potentially prevent the leading ribosome from being in close proximity to the RNA polymerase, which may increase the probability of transcription termination for some genes (86–88). The increased transcription termination would further enhance gene silencing by sRNAs. For sRNAs that increase target gene expression via opening up hairpins at the TIR (2) to facilitate ribosome binding, the capacity to enter the nucleoid and bind during the early stages of transcription would be expected to further enhance their activity by preventing transcription termination. Our demonstration that short lengths of mRNA can move through the nucleoid suggests that partial length mRNAs that are generated during transcription termination (88–90) can easily diffuse out of the nucleoid. This movement of partial length mRNAs will reduce entropic forces acting to expand the nucleoid and allow mRNA fragments to be quickly broken down and recycled by the RNA degradosome at the inner membrane (16,91).

During stress conditions and slow growth rates, the nucleoid can become more compact resulting in less space between the folded DNA and consequently greater resistance to the diffusion of large molecules through the nucleoid (38). Therefore, while we found that RNAs of 442 nucleotides or less in length were able to localize in the nucleoid, this may not be the case during stress, which may explain why sRNAs are much shorter (50–250 nucleotides) (92). The effect of stress on nucleoid localization of sRNAs and mRNAs needs to be further investigated and should be kept in mind when designing synthetic circuits.

Our findings are relevant in many ways for the design of synthetic gene circuits incorporating sRNAs and other non-coding RNAs. They directly demonstrate that the construction of synthetic gene circuits with sRNAs on plasmids will not impair these sRNAs from accessing the nucleoid and regulating target genes on the chromosome. In addition, we found little difference in the nucleoid localization of RNAs over a wide range of sizes from 102 nucleotides (RyhB) to 442 nucleotides (partial length *gfp* mRNA without RBS). Therefore synthetic sRNAs should be designed to be less than 442 nucleotides (or an R_g_ < ≈ 80 Å or a diameter < ≈ 200 Å), and probably shorter if they need to function during stress conditions for the reasons mentioned above. This constraint on size may limit the use of long non-coding RNAs, which are typically cis-acting and bind to complementary target mRNAs (10), particularly for applications where they need to act within the nucleoid to be efficient. Within the range of 442 to 1185 nucleotides it appears that as the RNA becomes larger it has more difficulty entering the nucleoid; this relationship between size and nucleoid localization needs to be further characterized. It must be stressed that size is not the only factor that may affect RNA localization. As we showed with the *bglF::gfp* mRNA, and others have shown for other RNAs (22), specific sequences can affect RNA localization, which could conceivably affect nucleoid localization of sRNAs.

Another point that is relevant to synthetic biology is the effect of localization on local RNA concentrations. Because sRNAs we investigated do not appear to sequester or concentrate in any specific regions of the cell their concentrations are simply determined by the whole volume of the cell. In contrast, mRNAs such as *gfp* (as well as *ptsG* and *bglF*) occupy a smaller volume because of exclusion from the nucleoid and therefore have higher local concentrations. Estimates of the volume of the nucleoid range from ∼50 to 75% of the cell volume (37,93), which means that with the same number of sRNA and mRNA molecules, the effective cytoplasmic mRNA concentration (if the mRNA is excluded from 75% of the cell volume) may be four times higher than the sRNA concentration. This difference is important in quantitative models of gene regulation, particularly for sRNAs due to their stoichiometric action (44,94) and for mRNAs encoding cooperative proteins and other proteins with steep response curves (83,84). Another consequence for the modeling of sRNAs that enter the nucleoid and therefore have greater potential for silencing, is that this is expected to alter several aspects of their threshold-linear response (42,44) (i.e. target protein concentration as a function of sRNA production) (Supplementary Figure S5). Specifically, it is expected that: (i) the linear graded response will be ‘steeper’ because each sRNA prevents more target proteins from being produced; (ii) the transition at the threshold will be sharper (44); and (iii) the minimum target protein concentration will be lower (Supplementary Figure S5).

In conclusion, this study reveals that sRNAs can move into the nucleoid and because of this they have the potential to regulate mRNAs deep within the nucleoid, soon after mRNA transcription is initiated and the TIR is synthesized, and before the transcription-translation complex moves to the edge of nucleoid. Furthermore, sRNAs appear to occur with equal probability in the nucleoid and cytoplasm which suggests there is no bias or sequestration of sRNAs in either region. This information provides a deeper understanding of the potential roles for sRNAs in gene regulation and of the potential constraints on the evolution of sRNAs, and allows the construction of more accurate and more detailed models to optimize the engineering of synthetic circuits incorporating sRNAs.

## Supplementary Material

Supplementary Data
