# Supplementary material for: Nucleoid and cytoplasmic localization of small RNAs in Escherichia coli
Source: Nucleic Acids Res. 2017 Jan 24;45(5):2919–34. doi: 10.1093/nar/gkx023 (PMC5389542; doi:10.1093/nar/gkx023)
Supplement: Supplementary Data [file gkx023_Supplementary_Data.zip › nar-02127-y-2016-File008.pdf]

SUPPLEMENTARY INFORMATION

Nucleoid and cytoplasmic localization of small RNAs in *Escherichia coli*

Huanjie Sheng[1], Weston T. Stauffer[1], Razika Hussein[1], Chris Lin[1], and Han N. Lim[1,2]

[1] Department of Integrative Biology, University of California Berkeley

[2] Corresponding author: drhanlim@gmail.com

SUPPLEMENTARY METHODS

RNA fluorescent in situ hybridization (RNA FISH)

RNA FISH was performed according to a published protocol (1) with the exception of modifications made to reduce the cost or increase signal as described in the main text.

The first part of the process of RNA FISH was growing the cells and fixing them. Cells from an overnight culture were inoculated into fresh lysogeny broth (LB) media with 100 µg/mL of ampicillin (and 1 mM IPTG for the *gfp* mRNAs). The cells were grown at 37°C and 200 revolutions per minute (rpm) on a shaker for 3.5 - 5 hours to an OD_{600nm} ~ 0.3-0.5. Then 7.5 mL of the culture was removed and centrifuged for 10 minutes at 4°C and 3650 g, the supernatant was removed and the cell pellet was resuspended in 0.5 mL of fixation solution [3.7% formaldehyde (Mallinckrodt Chemicals, Phillipsburg, NJ, USA) in 1 x PBS (phosphate buffered saline)] and incubated at 25°C for 30 minutes at 30 rpm. The fixed cells were centrifuged at 25°C for 8 minutes at 400 g, the supernatant was removed, and the cell pellet resuspended in 1 x PBS. The cells were then centrifuged at 25°C for 3.5 min at 600g and resuspended in 1 x PBS and this was repeated. The cells were resuspended in 70% ethanol and incubated at 25°C for 1 h to permeabilize them, and then centrifuged at 25°C for 7 minutes at 600 g, and the cell pellet resuspended in wash solution [35.43% formamide (Fisher Scientific, Fair Lawn, NJ, USA) in 2 x saline sodium citrate (SSC)] (1).

The second part of the process of RNA FISH was probe hybridization and DNA staining. Fixed cells in wash solution were centrifuged at 25°C for 7 minutes at 600 g and resuspended in 25 µl hybridization solution [10% w/v dextran sulfate (Pharmacia; now part of GE Healthcare Life Sciences), 2x SSC, 10% formamide, 2 mM ribonucleoside vanadyl complex (New England Biolabs, Ipswich, MA, USA), 200 µg/mL of bovine serum albumin (Sigma, St. Louis, MO, US), and 1 mg/mL of tRNA from *E. coli* MRE600 (Roche Diagnostics, Indianapolis, IN, USA)]. Note: we found the hybridization solution became unstable after multiple uses and therefore it was stored as single-use aliquots. Two µl of each fluorescently labeled probe at a concentration of 25 µM was added to each 25 µl hybridization solution (final volume is 27 µl) at 30°C overnight at 30 rpm

on a shaker. The next morning the probe was washed away as previously described (1). In the final wash step, 4', 6-diamidino-2-phenylindole (DAPI; Invitrogen, Grand Island, NY, USA) was added to a final concentration of 10 µg/mL to stain the DNA and incubated at 30ºC for 30 minutes and then centrifuged at 25ºC for 3.5 minutes at 600 g. After the final wash, the cells were resuspended in 100 µl of GLOX buffer (0.4% glucose, 10mM Tris-HCL and 2 x SSC) and incubated at 25ºC for 5 minutes. The cells were then centrifuged for 3.5 minutes at 600 g and resuspended in 10 µl of GLOX buffer with a final concentration of 0.43 mg/ml of bovine catalase (Sigma) and 1% glucose oxidase from *Aspergillus niger* (Sigma), placed on slides with coverslips and examined immediately by fluorescence microscopy.

Calculating the fraction of GlmZ and SgrS sRNAs bound to Hfq

We observed that the deletion of *hfq* decreased the signal for GlmZ and SgrS by approximately 50% (main text). This finding is consistent with previous reports that the binding of Hfq to these sRNAs decreases their degradation rates (2,3), and therefore the deletion of *hfq* will decrease their concentrations. From our finding we estimated the fraction of GlmZ and SgrS that are bound to Hfq using sets of equations to create a model of sRNA production and degradation (both in the presence and absence of Hfq). These equations are simplified from our previously reported models (3,4).

The model has a constant production rate P (units: $M \cdot s^{-1}$) because the same promoter and plasmid is used in strains with and without *hfq*. The degradation of the unbound sRNAs is proportional to their concentration U (unit: M) and is specified by the rate constant γ_U (units: s^{-1}). Unbound sRNA can bind Hfq to form a bound form with concentration H (unit: M). The rate constants for the sRNA and Hfq binding and unbinding reactions are k_U (units: $M^{-1} \cdot s^{-1}$) and k_H (unit: s^{-1}) respectively. The degradation of sRNAs bound to Hfq is also proportional to their concentration and is specified by the rate constant γ_H (unit: s^{-1}).

In the presence of Hfq, the equations for the system are:

$$\frac{dU}{dt} = P + k_H \cdot H \cdot Hfq - k_U \cdot U - \gamma_U \cdot U, \text{ and} \qquad [S1]$$

$$\frac{dH}{dt} = - k_H \cdot H \cdot Hfq + k_U \cdot U - \gamma_H \cdot H. \qquad [S2]$$

In our experiments we measured the total sRNA concentration by RNA FISH, which is U + H, therefore we combine the above equations to give

$$\frac{d(U+H)}{dt} = P + -\gamma_U \cdot U - \gamma_H \cdot H. \quad \text{[S3]}$$

At steady state,

$$P = \gamma_U \cdot U + \gamma_H \cdot H. \quad \text{[S4]}$$

In the absence of Hfq, bound sRNA does not occur therefore the equation for the system is:

$$\frac{dU}{dt} = P - \gamma_U \cdot U. \quad \text{[S5]}$$

At steady state,

$$P = \gamma_U \cdot U^*. \quad \text{[S6]}$$

* indicates the steady state concentration of unbound sRNA is not necessarily the same in the systems with Hfq and without Hfq (**Equation S4**).

For both GlmZ and SgrS, the total sRNA concentration in the *hfq* deletion mutant, which has only unbound sRNA, is approximately half that of the wild-type with *hfq*. That is,

$$U^* = \frac{1}{2}(U + H). \quad \text{[S7]}$$

The substitution of **Equation S7** into **Equation S6,** and using the equality of the right hand sides of **Equations S4** and **S6** gives

$$\gamma_U \cdot \frac{1}{2}(U + H) = \gamma_U \cdot U + \gamma_H \cdot H. \quad \text{[S8]}$$

Rearranging **Equation S8** specifies the ratio of U and H in terms of the degradation constants and incorporates the constraint obtained from the experimental observations (defined in **Equation S7**). That is,

$\frac{U}{H} = 1 - \frac{2\gamma_H}{\gamma_U}$, where $H > 0$ and $\gamma_U > 0$. [S9]

Biologically U and H must be greater than or equal to zero. We set aside the case where H is equal to zero for the purpose of interpreting **Equation S9** to avoid the ratio of U/H being undefined and also because it is already considered in **Equations S5** and **S6** for the *hfq* deletion strain. Consequently,

$1 - \frac{2\gamma_H}{\gamma_U} \geq 0$, where $\gamma_U > 0$. [S10]

Therefore,

$\frac{\gamma_H}{\gamma_U} \leq \frac{1}{2}$, where $\gamma_U > 0$. [S11]

Because $\gamma_H \geq 0$ and $\gamma_U > 0$ then

$0 \leq \frac{\gamma_H}{\gamma_U} \leq \frac{1}{2}$. [S12]

We now consider the limits of the $\frac{\gamma_H}{\gamma_U}$ ratio. The lower limit, $\frac{\gamma_H}{\gamma_U} = 0$, occurs when degradation of bound sRNA is zero and only unbound sRNAs are degraded. Under these conditions, **Equation S9** indicates the U/H ratio must be 1/1 to satisfy our experimental observations for GlmZ and SgrS. That is, approximately 50% of the sRNA are bound to Hfq when there is no degradation of Hfq bound sRNAs. Therefore the deletion of *hfq* and elimination of bound sRNAs would decrease the total sRNA concentration by 50%, as observed for GlmZ and SgrS. The upper limit, $\frac{\gamma_H}{\gamma_U} = \frac{1}{2}$, occurs when the value for the degradation rate constant for bound sRNAs is one half that of unbound sRNAs. In this scenario, the wild-type strain has a concentration of unbound sRNA that is essentially zero because sRNAs rapidly and stably bind to Hfq. Bound sRNAs have a concentration that is twice that of unbound sRNAs (the latter occur when *hfq* is deleted), because the degradation rate for bound sRNAs is half that of unbound sRNAs. The $\frac{\gamma_H}{\gamma_U}$ ratio cannot exceed 1:2 (*e.g.* 1:1) otherwise the total sRNA concentration in the wild-type strain is less than two-fold the concentration in the *hfq* deletion strain, and this would not be consistent with our measurements.

In summary, the 50% decrease in total sRNA concentration of GlmZ and SgrS with the deletion of *hfq* indicates that 50%-100% of these sRNAs are bound to Hfq.

SUPPLMENTARY REFERENCES

1. Zong, C., So, L.H., Sepulveda, L.A., Skinner, S.O. and Golding, I. (2010) Lysogen stability is determined by the frequency of activity bursts from the fate-determining gene. *Mol Syst Biol*, **6**, 440.
2. Adamson, D.N. and Lim, H.N. (2011) Essential requirements for robust signaling in Hfq dependent small RNA networks. *PLoS Comput Biol*, **7**, e1002138.
3. Hussein, R. and Lim, H.N. (2012) Direct comparison of small RNA and transcription factor signaling. *Nucleic Acids Res*, **40**, 7269-7279.
4. Adamson, D.N. and Lim, H.N. (2013) Rapid and robust signaling in the CsrA cascade via RNA-protein interactions and feedback regulation. *Proc Natl Acad Sci U S A*, **110**, 13120-13125.
5. Hussein, R. and Lim, H.N. (2011) Disruption of small RNA signaling caused by competition for Hfq. *Proc Natl Acad Sci U S A*, **108**, 1110-1115.
6. Shin, J.E., Lin, C. and Lim, H.N. (2016) Horizontal transfer of DNA methylation patterns into bacterial chromosomes. *Nucleic Acids Res*, **44**, 4460-4471.
7. Zhang, J., Jones, C.P. and Ferre-D'Amare, A.R. (2014) Global analysis of riboswitches by small-angle X-ray scattering and calorimetry. *Biochim Biophys Acta*, **1839**, 1020-1029.
8. Henderson, C.A., Vincent, H.A., Stone, C.M., Phillips, J.O., Cary, P.D., Gowers, D.M. and Callaghan, A.J. (2013) Characterization of MicA interactions suggests a potential novel means of gene regulation by small non-coding RNAs. *Nucleic Acids Res*, **41**, 3386-3397.
9. Muller, J.J., Zalkova, T.N., Zirwer, D., Misselwitz, R., Gast, K., Serdyuk, I.N., Welfle, H. and Damaschun, G. (1986) Comparison of the structure of ribosomal 5S RNA from E. coli and from rat liver using X-ray scattering and dynamic light scattering. *Eur Biophys J*, **13**, 301-307.
10. Noriega, T.R., Chen, J., Walter, P. and Puglisi, J.D. (2014) Real-time observation of signal recognition particle binding to actively translating ribosomes. *Elife*, **3**.
11. Kilburn, D., Roh, J.H., Behrouzi, R., Briber, R.M. and Woodson, S.A. (2013) Crowders perturb the entropy of RNA energy landscapes to favor folding. *J Am Chem Soc*, **135**, 10055-10063.
12. Lipfert, J., Das, R., Chu, V.B., Kudaravalli, M., Boyd, N., Herschlag, D. and Doniach, S. (2007) Structural transitions and thermodynamics of a glycine-dependent riboswitch from Vibrio cholerae. *J Mol Biol*, **365**, 1393-1406.
13. Peng, Y., Curtis, J.E., Fang, X. and Woodson, S.A. (2014) Structural model of an mRNA in complex with the bacterial chaperone Hfq. *Proc Natl Acad Sci U S A*, **111**, 17134-17139.
14. Kazantsev, A.V., Rambo, R.P., Karimpour, S., Santalucia, J., Jr., Tainer, J.A. and Pace, N.R. (2011) Solution structure of RNase P RNA. *RNA*, **17**, 1159-1171.
15. Gopal, A., Zhou, Z.H., Knobler, C.M. and Gelbart, W.M. (2012) Visualizing large RNA molecules in solution. *RNA*, **18**, 284-299.
16. Osterberg, R., Sjoberg, B., Garrett, G.A. and Muller, R. (1980) The conformation of a large RNA fragment from the E.coli ribosomal 16S-RNA. An X-ray and neutron small-angle scattering study. *Nucleic Acids Res*, **8**, 6221-6231.
17. Folkhard, W., Pilz, I., Kratky, O., Garrett, R. and Stoffler, G. (1975) Small-angle x-ray studies on the structure of 16-S ribosomal RNA and of a complex of ribosomal protein S4 and 16-S ribosomal RNA from Escherichia coli. *Eur J Biochem*, **59**, 63-71.
18. Stanley, W.M., Jr. and Bock, R.M. (1965) Isolation and physical properties of the ribosomal ribonucleic acid of Escherichia coli. *Biochemistry*, **4**, 1302-1311.
19. Mandiyan, V., Tumminia, S.J., Wall, J.S., Hainfeld, J.F. and Boublik, M. (1991) Assembly of the Escherichia coli 30S ribosomal subunit reveals protein-dependent folding of the 16S rRNA domains. *Proc Natl Acad Sci U S A*, **88**, 8174-8178.
20. Zipper, P. and Folkhard, W. (1975) A small-angle x-ray scattering investigation on the structure of the RNA from bacteriophage MS2. *FEBS Lett*, **56**, 283-287.
21. Werner, A. (2011) Predicting translational diffusion of evolutionary conserved RNA structures by the nucleotide number. *Nucleic Acids Res*, **39**, e17.

SUPPLEMENTARY FIGURE LEGENDS

Fig. S1. Plasmid maps. (**A**) pHL1391 and its derivatives (pHL1892, pHL1966, and pHL2000). pHL1391 was modified by replacing *rpoS* with either RBS(st7)::*bglF* or *fhlA*, and inserting an Asp terminator between the *Bam*HI and *Apa*I restriction sites to generate pHL1892 or pHL1966 respectively. pHL2000 is the same as pHL1391 except the partial *rpoS* sequences is replaced by the partial *sodB* sequence, and PCon (no *Hin*dIII site)::*ryhB* is inserted between *Bam*HI and *Apa*I in the same clockwise direction as *gfp*. (**B**) pHL1990 and its derivatives (pHL1991, pHL2004 and pHL2013). *pHL1990 plasmid was modified by replacing PCon (no *Hin*dIII site)::*dsrA* with: (i) PCon (no *Hin*dIII site)::*ryhB* generating pHL1991; (ii) PCon::*oxyS* generating pHL2004; (iii) PCon (no *Hin*dIII site)::*sgrS* generating pHL2013. (**C**) pHL2016. (**D**) pHL2017 and its derivatives (pHL2135, pHL2137, pHL2138 and pHL2139). *pHL2017 was modified by replacing the RBS(st7)::*gfp* with: (i) *gfp* (no ATG or RBS(st7)) generating pHL2135; (ii) first quarter RBS(st7) *gfp* generating pHL2137; (iii) last quarter *gfp* (no ATG or RBS(st7)) generating pHL2138; (iiii) first quarter *gfp* (no ATG or RBS(st7)) generating pHL2139. (**E**) pHL2095 and its derivatives (pHL2096, pHL2097 and pHL2099). *pHL2095 was modified by replacing *ryhB* with: (i) *oxyS* to generate pHL2096; (ii) *sgrS* generating pHL2097; (iii) *glmZ* generating pHL2099. (**F**) pHL2098.

Fig. S2. RNA FISH for DsrA and SgrS. Representative cells transcribing DsrA and SgrS with Cy5 labeled probes. White line is the cell boundary identified by phase contrast, which after alignment was transferred to the Cy5 channels. Yellow scale bar indicates 1 µm. The intensity of Cy5 signal in cells is shown as heat maps with "global normalization" (described in main text). Strains: *dsrA* (HL6269) with PCon promoter; *sgrS* with PCon promoter (HL6332; data from **Fig.1A** is reshown for comparison).

Fig. S3. Top 10% of signal for sRNAs, mRNA or DNA in cells. (**A**) sRNA and mRNA localization in representative cells (same as shown in **Figure 2A**) transcribing sRNAs or mRNAs in phase contrast, DAPI, and Cy5 or Cy3 channels. Pixels with highest 10% of DAPI signal (DNA) are colored cyan and pixels with highest 10% of Cy5 signal (sRNA) or highest 10% of Cy3 signal (mRNA) are colored magenta. Any pixels that have the highest 10% of DAPI signal and the highest 10% of Cy3 or Cy5 signal (*i.e.* overlapping pixels)

are colored yellow. Note: readers may need to use zoom on the images to see individual pixels. Yellow scale bar indicates 1 μm. Strains are described in the legend for **Figure 2A**. (**B**) Representative cells (same as shown in **Figure 2B**) in the negative control (HL716; *n* = 355) without Cy3 or Cy5 probes in phase contrast, DAPI, Cy3 and Cy5 channels. Pixels are colored as described in **panel A**. Yellow scale bar indicates 1 μm.

Fig. S4. Histograms of membrane TOS. The data is from the experiment shown in Fig. 3. All histograms have the same bin width of 0.1 TOS (unitless). Grey dash line indicates membrane TOS = 0.

Fig. S5. Expected threshold-linear responses for sRNAs that can and cannot enter the nucleoid with all other factors being equal. The sRNAs are decreasing translation of the target mRNA.

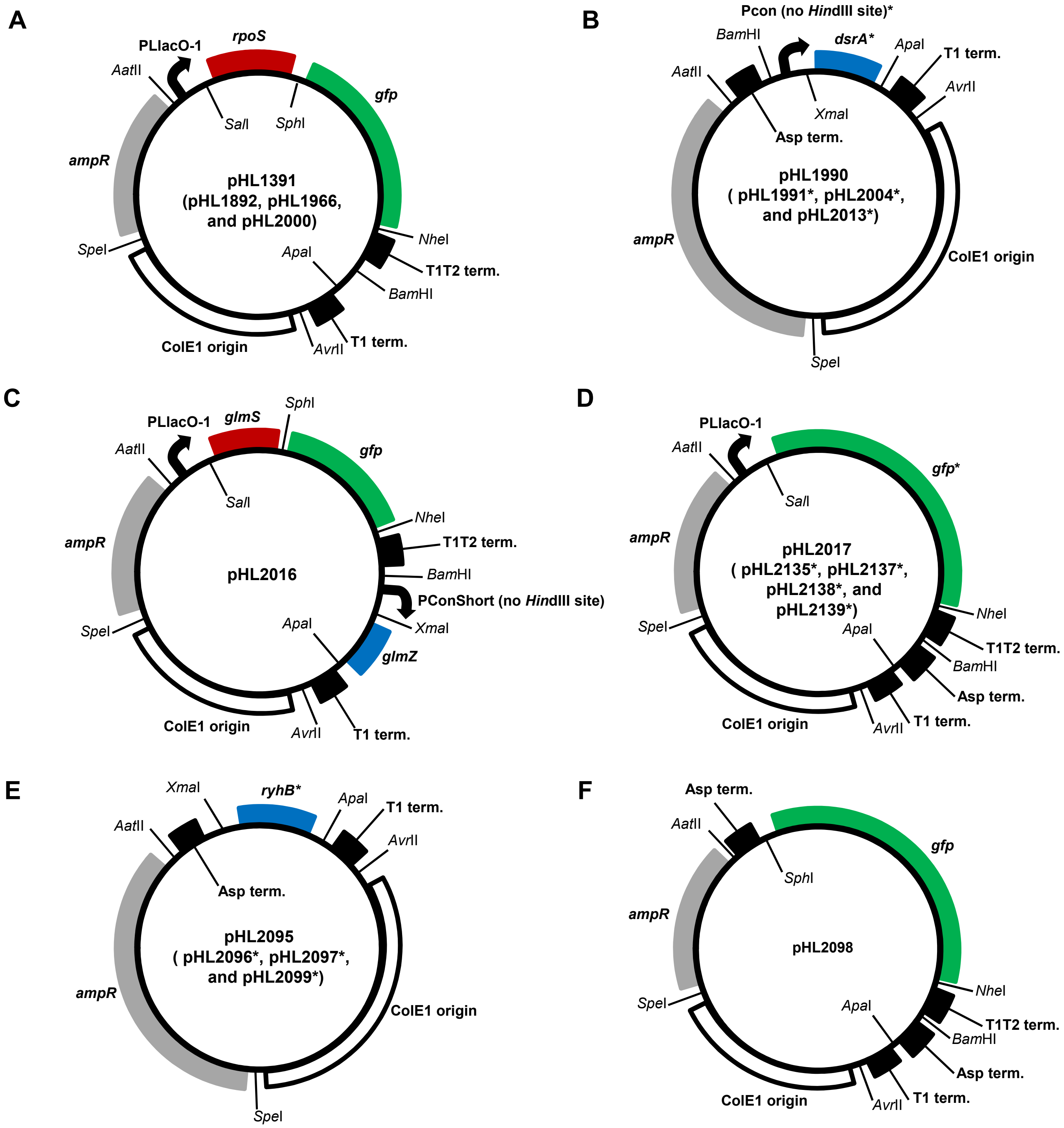
A
PLlacO-1
rpoS
AatII
gfp
SalI
SphI
ampR
pHL1391
(pHL1892, pHL1966,
and pHL2000)
NheI
SpeI
ApaI
T1T2 term.
BamHI
ColE1 origin
AvrII
T1 term.
B
Pcon (no HindIII site)*
BamHI
dsrA*
ApaI
T1 term.
AatII
AvrII
XmaI
Asp term.
pHL1990
(pHL1991*, pHL2004*,
and pHL2013*)
ampR
ColE1 origin
SpeI
C
PLlacO-1
glmS
SphI
AatII
gfp
SalI
NheI
ampR
T1T2 term.
pHL2016
BamHI
PConShort (no HindIII site)
SpeI
ApaI
XmaI
glmZ
ColE1 origin
AvrII
T1 term.
D
PLlacO-1
AatII
gfp*
SalI
ampR
pHL2017
(pHL2135*, pHL2137*,
pHL2138*, and
pHL2139*)
NheI
SpeI
ApaI
T1T2 term.
BamHI
Asp term.
ColE1 origin
AvrII
T1 term.
E
XmaI
ryhB*
ApaI
T1 term.
AatII
AvrII
Asp term.
pHL2095
(pHL2096*, pHL2097*,
and pHL2099*)
ampR
ColE1 origin
SpeI
F
Asp term.
AatII
gfp
SphI
ampR
pHL2098
NheI
SpeI
ApaI
T1T2 term.
BamHI
Asp term.
ColE1 origin
AvrII
T1 term.

Fig. S1.

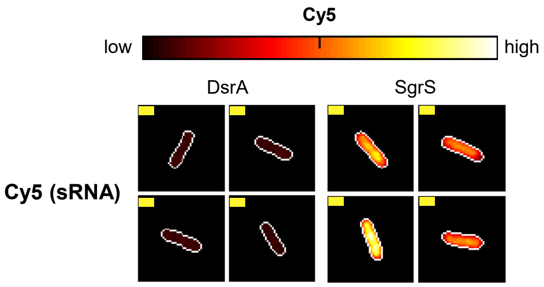

Fig. S2.

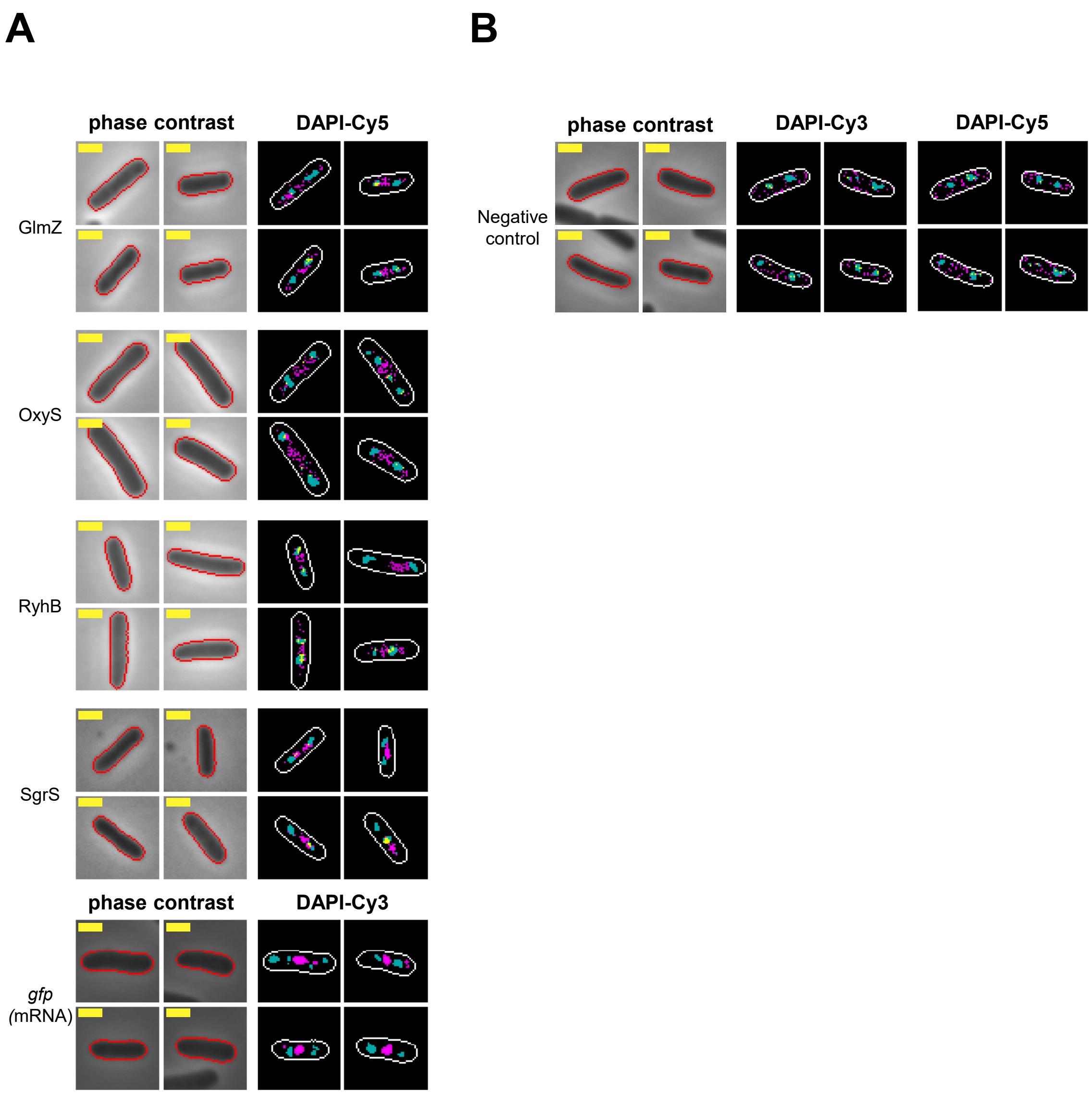
A
B
phase contrast
DAPI-Cy5
GlmZ
OxyS
RyhB
SgrS
phase contrast
DAPI-Cy3
gfp
(mRNA)
phase contrast
DAPI-Cy3
DAPI-Cy5
Negative
control

Fig. S3.

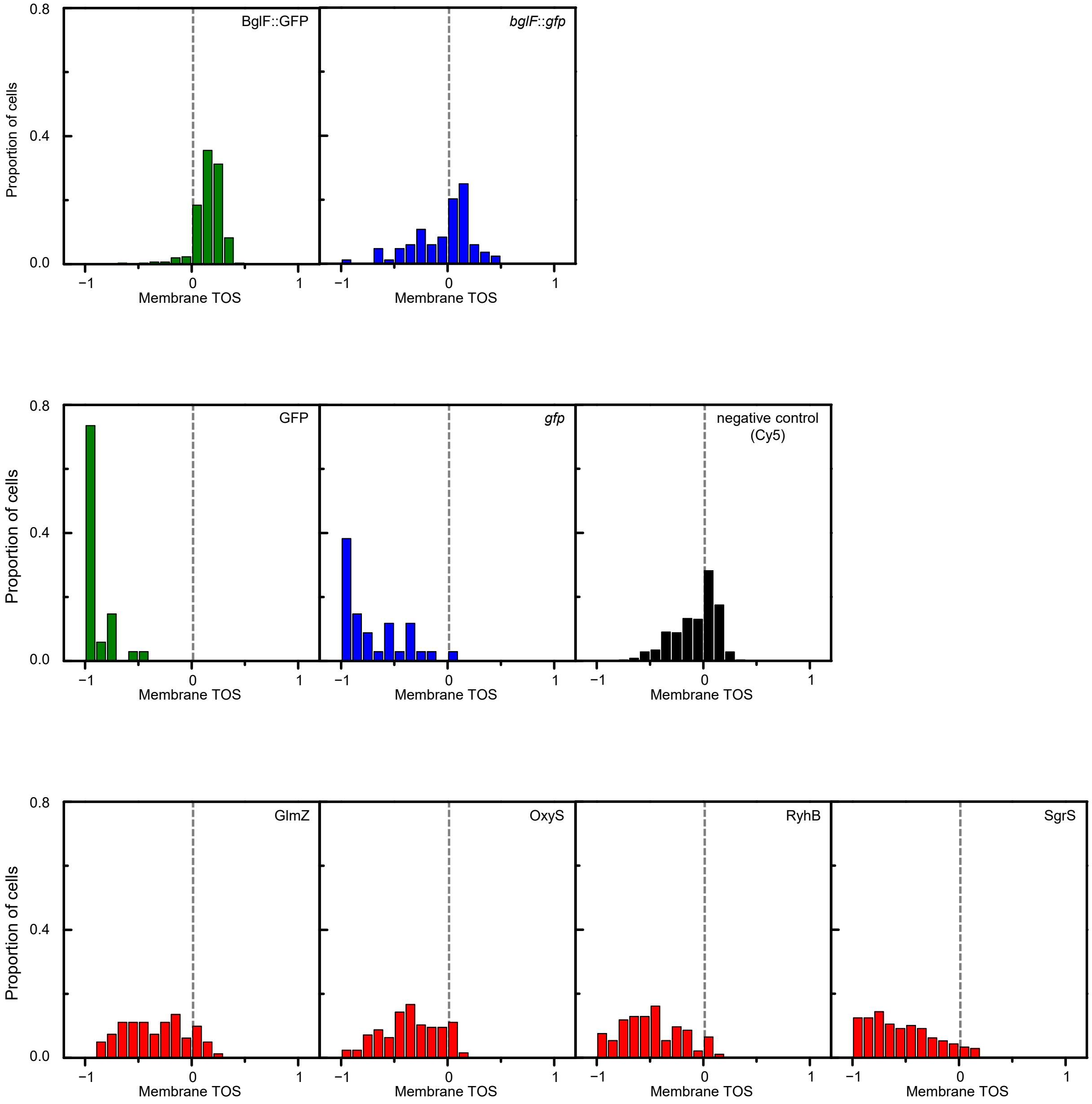
0.8
0.4
0.0
Proportion of cells
BglF::GFP
bglF::gfp
−1
0
1
Membrane TOS
GFP
gfp
negative control
(Cy5)
GlmZ
OxyS
RyhB
SgrS

Fig. S4.

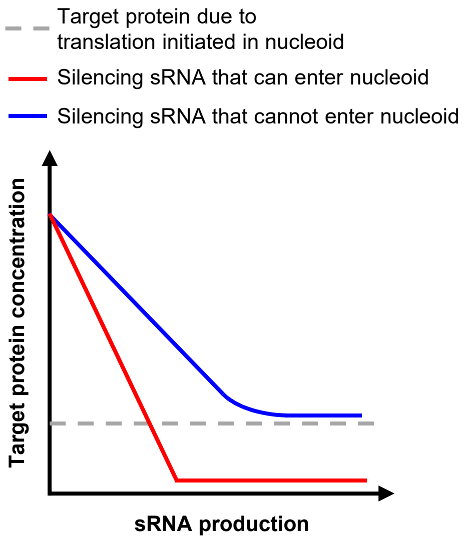
Target protein due to
translation initiated in nucleoid
Silencing sRNA that can enter nucleoid
Silencing sRNA that cannot enter nucleoid
Target protein concentration
sRNA production

Fig. S5.

Table S1. Strains.

Strain	Description	Source	Antibiotic Resistance
HL1	MG1655 + pKD46	(5)	amp
HL713	HL1 + integration at *intS* of PCR product *kanR::lacIq* amplified from pHL67 with intspkd1f and laciqints	This study	kan
HL716	HL713 + pCP20 and cured	(5)	none
HL744	HL716 + pKD46	(6)	amp
HL751	HL744 + Δ*hfq* using pKD13 and oligonucleotides hfqpkd1f and hfqpkd4r	This study	kan
HL752	HL744 + Δ*sgrS* using pKD13 and oligonucleotides sgrsko1pkd1f and sgrsko2pkd4r	(6)	kan
HL756	HL752 + pCP20 and cured	This study	none
HL770	HL751 + pCP20 and cured	This study	none
HL772	HL770 + pKD46	This study	amp
HL852	HL744 + Δ*dsrA* using pKD13 and oligonucleotides dsrako1pkd1f and dsrako2pkd4r	This study	kan
HL865	HL852 + pCP20 and cured	(5)	none
HL2729	HL744 + Δ*ryhB* using pKD13 and oligonucleotides rybpkd1f and rybpkd4r	This study	kan
HL2752	HL2729 + pCP20 and cured	(5)	none
HL3221	HL744 + Δ*oxyS* using pKD13 and oligonucleotides oxys1pkd1f and oxys2pkd4r	This study	kan
HL3262	HL3221 + pCP20 and cured	(5)	none
HL3325	HL772 + Δ*ryhB* using pKD13 and oligonucleotides rybpkd1f and rybpkd4r	This study	kan
HL3338	HL3325 + pCP20 and cured	(5)	none
HL3387	HL772 + Δ*oxyS* using pKD13 and oligonucleotides oxys1pkd1f and oxys2pkd4r	This study	kan
HL3425	HL3387 + pCP20 and cured	(5)	none
HL5212	HL744 + Δ*glmZ* using pKD13 and oligonucleotides glmzkopkd1f and glmzkopkd4r	This study	kan
HL5226	HL5212 + pCP20 and cured	This study	none
HL5316	HL5226 + pKD46	This study	amp
HL5378	HL5316 + Δ*glmY* using pKD13 and oligonucleotides glmykopkd1f and glmykopkd4r	This study	kan
HL5390	HL5378 + pCP20 and cured	This study	none
HL5969	HL716 + pHL1892	This study	amp
HL6040	HL744 + Δ*yhbJ* using pKD13 and oligonucleotides yhbjshortpkd1f and yhbjshortpkd4r	This study	kan
HL6128	HL5390 + Δ*hfq* via transduction from HL6040	This study	kan
HL6190	HL6128 + pCP20 and cured	This study	amp
HL6193	HL716 + pHL1391	(5)	amp
HL6201	HL716 + pHL1966	This study	amp
HL6268	HL2752 + pHL1991	This study	amp
HL6269	HL865 + pHL1990	This study	amp
HL6284	HL2752 + pHL2000	This study	amp
HL6285	HL3338 + pHL2000	This study	amp
HL6286	HL3338 + pHL1991	This study	amp
HL6317	HL756 + Δ*hfq* via transduction from HL751	This study	kan
HL6318	HL3262 + pHL2004	This study	amp
HL6319	HL3425 + pHL2004	This study	amp
HL6320	HL5390 + pHL2016	This study	amp
HL6321	HL6190 + pHL2016	This study	amp
HL6322	HL716 + pHL2017	This study	amp
HL6332	HL752 + pHL2013	This study	(kan) amp
HL6333	HL6317 + pHL2013	This study	(kan) amp
HL6530	HL2752 + pHL2095	This study	amp
HL6531	HL3262 + pHL2096	This study	amp
HL6532	HL752 + pHL2097	This study	(kan) amp
HL6533	HL716 + pHL2098	This study	amp
HL6547	HL5390 + pHL2099	This study	amp
HL6733	HL716 + pHL2135	This study	amp
HL6735	HL716 + pHL2137	This study	amp
HL6736	HL716 + pHL2138	This study	amp
HL6737	HL716 + pHL2139	This study	amp

(kan) = kanamycin resistance but kanamycin not used for selection in the experiment

Table S2. Plasmids.

Plasmid	Description	Source	Antibiotic Resistance
pHL67	*lacIq* from pTrc99a + ColE1 from pZE21 + *kanR* cassette from pKD13 (including P1 and P4 oligonucleotide sites); template for lacIq insertion into the genome	(5)	kan
pHL1391	*ampR* + PLlacO-1::*rpoS*::*gfp*::T1T2 terminator + T1 terminator + ColE1	(5)	amp
pHL1966	*ampR* + PLlacO-1::*fhlA*::*gfp*::T1T2 terminator + T1 terminator + ColE1	This study	amp
pHL1892	*ampR* + PLlacO-1::RBS (st7)::*bglF*::*gfp*::T1T2 terminator + Asp terminator + T1 terminator + ColE1	This study	amp
pHL1990	*ampR* + Asp terminator + PConNoHind::*dsrA*::T1 terminator + ColE1	This study	amp
pHL1991	*ampR* + Asp terminator + PConNoHind::*ryhB*::T1 terminator + ColE1	This study	amp
pHL2000	*ampR* + PLlacO-1::*sodB*::*gfp*::T1T2 terminator + PConNoHind::*ryhB*::T1 terminator + ColE1	This study	amp
pHL2004	*ampR* + Asp terminator + PCon::*oxyS*::T1 terminator + ColE1	This study	amp
pHL2013	*ampR* + Asp terminator + PConNoHind::*sgrS*::T1 terminator + ColE1	This study	amp
pHL2016	*ampR* + PLlacO-1::*glmS*::*gfp*::T1T2 terminator + PConShortNoHind::*glmZ*::T1 terminator + ColE1	This study	amp
pHL2017	*ampR* + PLlacO-1::RBS (st7) *gfp*::T1T2 terminator + Asp terminator + T1 terminator + ColE1	This study	amp
pHL2095	*ampR* + Asp terminator::*ryhB*::T1 terminator + ColE1	This study	amp
pHL2096	*ampR* + Asp terminator::*oxyS*::T1 terminator + ColE1	This study	amp
pHL2097	*ampR* + Asp terminator::*sgrS*::T1 terminator + ColE1	This study	amp
pHL2098	*ampR* + Asp terminator::*gfp*::T1T2 terminator + Asp terminator + T1 terminator + ColE1	This study	amp
pHL2099	*ampR* + Asp terminator::*glmZ*::T1 terminator + ColE1	This study	amp
pHL2135	*ampR* + PLlacO-1::*gfp* (no RBS, no ATG)::T1T2 terminator + Asp terminator + T1 terminator + ColE1	This study	amp
pHL2137	*ampR* + PLlacO-1::RBS (st7) *gfp* (first quarter)::T1T2 terminator + Asp terminator + T1 terminator + ColE1	This study	amp
pHL2138	*ampR* + PLlacO-1::*gfp* (last quarter, no RBS, no ATG)::T1T2 terminator + Asp terminator + T1 terminator + ColE1	This study	amp
pHL2139	*ampR* + PLlacO-1::*gfp* (first quarter, no RBS, no ATG)::T1T2 terminator + Asp terminator + T1 terminator + ColE1	This study	amp

Table S3. Oligonucleotides.

Forward	Reverse	Sequence	Function (strain created)
dsrako1pkd1f	dsrako2pkd4r	atatggcgaatattttcttgtcagcgaaaaaaattgcggataaggtgatggtgtaggctggagctgcttc	delete *dsrA* using pKD13 as template in HL852
dsrako2pkd4r	dsrako1pkd1f	tattcatgacttcagcgtctctgaagtgaatcgttgaatgcacaataaaaattccggggatccgtcgacc	delete *dsrA* using pKD13 as template in HL852
glmykopkd1f	glmykopkd4r	agttcagatacaacaaagccgggaattacccggctttgttatggaataaggtgtaggctggagctgcttc	delete *glmY* using pKD13 as template in HL5378
glmykopkd4r	glmykopkd1f	cgttaccaaactattttctttattggcacagttactgcataatagtaaccattccggggatccgtcgacc	delete *glmY* using pKD13 as template in HL5378
glmzkopkd1f	glmzkopkd4r	tagttccttctcacccggaggcaagcacctccggggccttcctgatacatgtgtaggctggagctgcttc	delete *glmZ* using pKD13 as template in HL5212
glmzkopkd4r	glmzkopkd1f	acaagtgttaagggatgttatttcccgattctctgtggcataataaacgaattccggggatccgtcgacc	delete *glmZ* using pKD13 as template in HL5212
hfqpkd1f	hfqpkd4r	tcagaatcgaaaggttcaaagtacaaataagcatataaggaaaagagagagtgtaggctggagctgcttc	delete *hfq* using pKD13 as template in HL751, HL1120, and HL1179
hfqpkd4r	hfqpkd1f	ggaacgcaggatcgctggctccccgtgtaaaaaaacagcccgaaaccttaattccggggatccgtcgacc	delete *hfq* using pKD13 as template in HL751, HL1120, and HL1179
intspkd1f	laciqints	ccgtagatttacagttcgtcatggttcgcttcagatcgttgacagccgcagtgtaggctggagctgcttc	PCR amplify *kanR::lacIq* using pHL67 as template to integrate at *intS*
laciqints	intspkd1f	atagttgttaaggtcgctcactccaccttctcatcaagccagtccgcccagctaactcacattaattgcgttgc	PCR amplify *kanR::lacIq* using pHL67 as template to integrate at *intS*
oxys1pkd1f	oxys2pkd4r	agcaatgaacgattatccctatcaagcattctgactgataattgctcacagtgtaggctggagctgcttc	delete *oxyS* using pKD13 as template in HL3221 and HL3387
oxys2pkd4r	oxys1pkd1f	atttatatgtataaatttgagcctggcttatcgccgggctttttttatggcattccggggatccgtcgacc	delete *oxyS* using pKD13 as template in HL3221 and HL3387
rybpkd1f	rybpkd4r	gattttgaggatggttgagagggttgcagggtagtagataagttttagatgtgtaggctggagctgcttc	delete *ryhB* using pKD13 as template in HL2729 and HL3325
rybpkd4r	rybpkd1f	tttgcacaaccgcagaacttttccgcagggcatcagtcttaattagtgccattccggggatccgtcgacc	delete *ryhB* using pKD13 as template in HL2729 and HL3325
sgrsko1pkd1f	sgrsko2pkd4r	gcaaaagacagcaattttattttccctatattaagtcaataattcctaacgtgtaggctggagctgcttc	delete *sgrS* using pKD13 as template in HL752
sgrsko2pkd4r	sgrsko1pkd1f	gccatcgtcattatccagatcatacgttcccttttttagcgcggcgagaatattccggggatccgtcgacc	delete *sgrS* using pKD13 as template in HL752
yhbjshortpkd1f	yhbjshortpkd4r	atgcccagcttgtttgtgatttcaacagtttgcttgacgggtgtaggctggagctgcttc	delete *yhbJ* using pKD13 as template in HL6040
yhbjshortpkd4r	yhbjshortpkd1f	cggtaatgtctcttttagacgttgtgaggagaaacagtacattccggggatccgtcgacc	delete *yhbJ* using pKD13 as template in HL6040

Table S4. FISH probe sequences.

Oligonucleotide	Fluorescence label	Sequences	Function
gfp	Quasar Cy3	aagttcttctcctttacgca<br>gaattgggacaactccagtg<br>acatcgccatctaattcaac<br>gacagagaatttttgcccat<br>catcaccttcaccctctcca<br>agggtaagttttccgtatgt<br>tcccagtagtgcaaataaat<br>gttggccatggaacaggtag<br>ataaccgaaagtagtgacaa<br>atctcgcaaagcattgaaca<br>tgctgtttcatatgatctgg<br>catggcactcttgaaaaagt<br>tttcctgtacataaccttcg<br>gttcccgtcatctttgtaaa<br>tgacttcagcacgtgtcttg<br>acaagggtatcaccttcaaa<br>accttttaactcgattctat<br>ttccatcttctttaaaatca<br>tccattttgtgtccaagaat<br>attatgtgagttatagttgt<br>gtttgtctgccatgatgtat<br>ttaactttgattccattctt<br>aatgttgtgtctaattttga<br>ctaattgaacgcttccatct<br>gtattttgttgataatggtc<br>gacagggccatcgccaattg<br>ggtaatggttgtctggtaaa<br>gaaagggcagattgtgtgga<br>tctcttttcgttgggatctt<br>actcaagaaggatcatgtga<br>gtaatcccagcagctgttac<br>tgtatagttcatccatgcca	probing for *gfp* mRNA
DsrA	Quasar Cy5	caccaggaaatctgatgtgt<br>gcttaagcaagaagcactta<br>tgagggggtcgggatgaaac	probing for DsrA sRNA
GlmZ	Quasar Cy5	gagatggaatgagcatctac<br>tgaggcactaaggcgaacat<br>ctctgcgtcattccggagtt<br>ggacgataagcaccgtaaac<br>ggcataagcgacatctgtca<br>ttgtgtccatggtgtctgat<br>caagtgggtgcttcactcaa<br>gcgttaaaacaggtctgtat<br>gcctgctcttattacggagc	probing for GlmZ sRNA
OxyS	Quasar Cy5	aagaggtgccgctccgtttc<br>gggcagtgacttcaagggtt<br>cgagttgagaaactctcgaa<br>gttcacgttggctttagtta<br>cggatcctggagatccgcaa	probing for OxyS sRNA
RyhB	Quasar Cy5	gcgagggtcttcctgatcgc<br>atgtcgtgctttcaggttct<br>aatactggaagcaatgtgag<br>gccagcacccggctggctaa	probing for RyhB sRNA
SgrS	Quasar Cy5	gggcaccccccttgcttcatc<br>gtgctgataaaactgacgca<br>acttcgctgtcgcggtaaaa<br>cttaaccaacgcaaccagca<br>catggttaatcgttgtggga<br>tcccactgcatcagtccttc<br>tcaactttcagaattgcggt<br>agtcacacatgatgcaggca<br>gggtgattttacaccaatac<br>ccagcaggtataatctgctg	probing for SgrS sRNA

Table S5. Parameter values for experimental data analyses.

Microscope type	Initial size filter ($pixel^2$)	Second size filter ($pixel^2$)	AR filter	MinFeret filter (pixels)	Radius 1 (pixels)	Radius 2 (pixels)
Nikon TE2000E	[300,1100]	[300,900]	(2.01, ∞)	[0,30]	25	50
Zeiss AxioObserver Z1 (optovar 1.0 ×)	[100,400]	[100,300]	(2.01, ∞)	[0,10]	25	50
Zeiss AxioObserver Z1 (optovar 1.6 ×)	[200,800]	[200,600]	(2.01, ∞)	[0,15]	25	50

"Initial size filter" displays the minimum and maximum particle areas selected before watershed segmentation. "Second size filter" displays the minimum and maximum particle areas selected after watershed segmentation. "AR filter" is the lower and upper bounds of the major to minor axis used to select shape. "MinFeret" is the lower and upper bounds of the minimum caliper diameter for cells. "Radius 1" and "Radius 2" are the rolling ball radii used in the "Subtract Background" function for phase-contrast and fluorescence images respectively.

Table S6. Radii of gyration for bacterial single stranded RNAs.

RNA	Length (nucleotides)	Radius of gyration# (Å)	Source
S-adenosyl methionine riboswitch (unbound)	51	25	(7)
S-adenosyl methionine riboswitch (unbound)	52	31.7	(7)
fragment from 5S ribosomal RNA	62	28.5, 28.7 [28.6]	PDB: 357D, 364D
MicA small RNA	75	33.9	(8)
tRNA (valine)	76	24.4	PDB: 2K4C
tRNA (fMet)	77	22.6	PDB: 3CW5
thiamine pyrophosphate riboswitch (unbound)	83	27.5	(7)
DsrA small RNA	87	43.2	(8)
S-adenosyl methionine riboswitch (unbound)	94	29.9	(7)
cyclic diguanylate riboswitch (unbound)	98	32	(7)
yybP-ykoY Mn riboswitch	107	34.6	PDB: 4Y1M
5S ribosomal RNA	120	32.7	(9)
5S ribosomal RNA	120	36.1	(10)
flavin mononucleotide riboswitch (unbound)	141	29.4	(7)
lysine riboswitch (unbound)	181	43	(7)
Ribozyme (*Azoarcus*)	195	~60	(11)
glycine riboswitch (unbound)	226	45	(12)
rpoS mRNA (partial sequence)	284	68.1	(13)
ribonuclease P ribozyme	400	43.9, 44.3, 48 [45.4]	(14)
random intergenic sequence transcribed	975	182	(15)
12S (partial 16S)	~1000	71	(16)
random intergenic sequence transcribed	1523	208	(15)
16S ribosomal RNA	1541	176	(17)
16S ribosomal RNA	1541	189, 161 [175]	(18)
16S ribosomal RNA	1541	114	(19)
mRNA sequence (cowpea chlorotic mottle virus)	2777	172	(15)
23S ribosomal RNA	2904	230	(18)
MS2 RNA (bacteriophage)	3569	181	(20)

§ Radius of gyration was calculated from structures deposited in The Nucleic Acid Database Project at Rutgers, The State University of New Jersey. # The hydrodynamic radius is assumed to be approximately the same for RNAs (21). * Classified as non-riboswitch and non-ribozyme RNA structures. Values in square brackets [ ] are the average.
